# Time-resolved sensing of electromagnetic fields with single-electron interferometry

**DOI:** 10.1038/s41565-025-01888-2

**Published:** 2025-03-17

**Authors:** H. Bartolomei, E. Frigerio, M. Ruelle, G. Rebora, Y. Jin, U. Gennser, A. Cavanna, E. Baudin, J.-M. Berroir, I. Safi, P. Degiovanni, G. C. Ménard, G. Fève

**Affiliations:** 1 Laboratoire de Physique de l’Ecole normale supérieure, ENS, Université PSL, CNRS, Sorbonne Université, Université Paris Cité, Paris, France; 2 Univ. Lyon, ENS de Lyon, Université Claude Bernard Lyon 1, CNRS, Laboratoire de Physique, Lyon, France; 3 Centre de Nanosciences et de Nanotechnologies (C2N), CNRS, Université Paris-Saclay, Palaiseau, France; 4 Laboratoire de Physique des Solides, CNRS, Université Paris-Saclay, Orsay, France

**Keywords:** Electronic devices, Quantum Hall

## Abstract

Characterizing quantum states of the electromagnetic field at microwave frequencies requires fast and sensitive detectors that can simultaneously probe the field’s time-dependent amplitude and its quantum fluctuations. So far, this has been achieved by using either homodyne detection or fast digitizers. Both methods rely on the extraction of microwave radiation through an amplification chain towards the detector placed at room temperature, thereby limiting the time resolution to the ~10-GHz bandwidth of the measurement chain. Additionally, the coupling of high-impedance samples to the 50-Ω measurement chain is very weak, setting strong limitations on the detection sensitivity. In this work, we demonstrate an on-chip quantum sensor that exploits the phase of a single-electron wavefunction, measured in an electronic Fabry–Pérot interferometer, to detect the amplitude of a classical time-dependent electric field. The interferometer is implemented in a GaAs/AlGaAs quantum Hall conductor. The time resolution, limited by the temporal width of the electronic wavepacket, is ~35 ps. The interferometry technique provides a voltage resolution of ~50 μV, corresponding to a few microwave photons. Importantly, our detector measures both phase and contrast of the interference pattern. The latter opens the way to the detection of non-classical electromagnetic fields, such as squeezed or Fock states.

## Abstract (web summary)

In an interferometer using the ballistic propagation of electrons in a quantum Hall conductor, the phase of a single-electron wavefunction can act as a sensor for the detection of fast electric fields of small amplitude.

## Main

Recently, tremendous progress has been made in the field of electron quantum optics^1,2^, aiming at the generation and manipulation of electronic quantum states propagating in nanoconductors. Single-electron sources^3^ have been implemented^4–8^ and their coherence properties have been characterized from two-particle interferometry^7,9,10^. Tomography protocols for the reconstruction of single-electron states have also been proposed^11,12^ and experimentally realized^13–15^. In the meantime, various electronic interferometers have been demonstrated^16–18^. In the context of electron quantum optics, interferometers can be used to characterize^19^ and manipulate^20^ quantum electronic states for the encoding and processing of quantum information^12^ and for the readout of quantum entanglement^21–23^. Long confined to very pure GaAs/AlGaAs heterostructures, these techniques are now developing rapidly in other materials such as graphene^24–27^. This shows that the field has reached the maturity level needed for the development of its applications in two main different directions: for the processing of quantum information encoded in electronic flying qubits^28,29^ and quantum sensing based on single-electron wavefunctions^30^.

In electron quantum optics, quantum sensing would exploit the quantum coherence of single-electron states for the detection of quantum objects, such as the quantum states of an electromagnetic field. Quantum radiation can be characterized by the relatively high frequency of the electromagnetic field (gigahertz and beyond) and by a small number of photon excitations.

The detection of a quantum field, thus, requires the use of fast and sensitive detectors. In addition, quantum states of the electromagnetic field, such as Fock or squeezed vacuum states, have a vanishing average field amplitude, such that all information is encoded in the field fluctuations. This imposes a huge challenge for the development of quantum detectors that would be both fast and sensitive and would simultaneously probe the amplitude and fluctuations of the electromagnetic field. The short temporal width (a few picoseconds) of single-electron states, which naturally points to the required short temporal resolution, has already been exploited for the picosecond sampling of a time-dependent voltage^31^. This first demonstration of a sensing application using single electrons relied on the time modulation of transmission probability through an energy-selective potential barrier^32^. This method did not exploit the quantum coherence of single-electron states, bringing limitations in terms of the sensitivity (of the order of a few hundred microvolts), but, more importantly, on the possibility to detect quantum states, as such a method would only probe the amplitude of the detected voltage and be insensitive to quantum fluctuations.

**Fig. 1:**
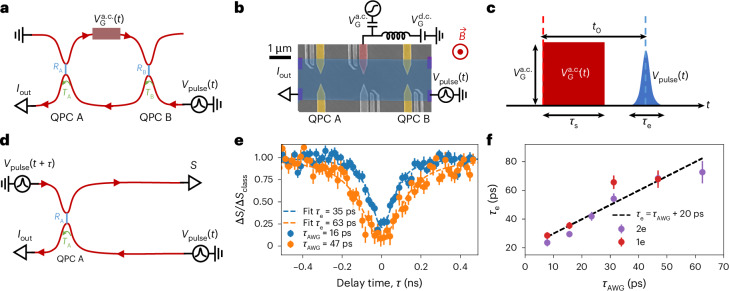
Principle of the experiment. **a**, QPC A and QPC B are used for partitioning of the outer-edge channel with transmission probabilities *T*_*i*_ = 1 − *R*_*i*_ (*i* = 1, 2), defining an electronic FPI. A single-electron pulse *V*_pulse_(*t*) is sent through the bottom-right branch of the interferometer. The square voltage ${V}_{{\rm{G}}}^{\rm{a.c.}}(t)$ we probe is imposed on the central gate located on the upper arm of the FPI. The d.c. current *I*_out_ is measured at the output of the FPI (bottom left). **b**, False-colour electron microscopy image of the sample. QPCs A and B are indicated in yellow. The interferometer is an FPI of height *H* = 2 ± 0.2 μm (taking into account a depletion length of 0.25 ± 0.1 μm on each side of the sample) and width *W* = 3.6 ± 0.2 μm. The perimeter of the FPI is *L* = 2 × (*H* + *W*) = 11.2 ± 0.8 μm and its area is *A* = 7.2 ± 0.8 μm^2^. The excitation gate (red) is connected through a bias-tee to a d.c. and a.c. source such that we can send both radio-frequency square excitation ${V}_{G}^{\rm{a.c.}}(t)$ and d.c. voltage ${V}_{\rm{G}}^{\rm{d.c.}}$. A magnetic field ${\bf{B}}$ perpendicular to the surface is applied to the sample. The two-dimensional electron gas mesa is indicated in blue and ohmic contacts are indicated in purple. All the other gates (grey) are not used in this experiment and are left floating. **c**, *V*_pulse_(*t*) is a single-electron Lorentzian pulse of width *τ*_e_. The square voltage ${V}_{G}^{\rm{a.c.}}(t)$ has a width *τ*_s_ and a peak-to-peak amplitude ${V}_{G}^{\rm{a.c.}}$. *t*_0_ is the time delay between ${V}_{G}^{\rm{a.c.}}(t)$ and *V*_pulse_*(t)*. **d**, In the HOM configuration, we probe the width of the Lorentzian pulses by measuring the current noise coming out of QPC A. **e**, Result of the HOM experiment showing the amplitude of noise (dots) as a function of the time difference between the two incoming Lorentzian pulses on QPC A for *τ*_AWG_ = 16 ps (blue) and *τ*_AWG_ = 47 ps (orange). The error bars represent the standard error of the noise measurements and are centred around its mean value. A fit of the data (in dotted lines) shows that the actual time widths at the level of the sample are 35 ps and 63 ps. **f**, Measured width of the pulses as a function of the set time on the AWG for pulses containing one (1e; red) or two (2e; purple) electrons. We observe a linear dependence of *τ*_e_ = *τ*_AWG_ + 20 ps. The error bars are extracted from a Lorentzian fit of the HOM noise data and represent one standard deviation (as shown in **e**).

In this work, we exploit, for the first time to our knowledge, the quantum coherence of single-electron states measured in an electronic Fabry–Pérot interferometer (FPI) for the readout of a fast time-dependent voltage applied on a gate placed in one arm of the interferometer (Fig. 1a). As already demonstrated in previous experiments^31^, the short temporal width of single-electron states exhibits a temporal resolution of a few tens of picoseconds. In addition, the interferometry technique we use here has a sensitivity of a few tens of microvolts. More importantly, the quantum nature of our detection scheme, where the detected field is directly imprinted in the phase of the electronic wavefunction, is naturally suited for the detection of quantum radiation. Using a classical voltage drive, we demonstrate here that the field amplitude can be directly extracted from the phase shift of the measured interference pattern. In future experiments, where the classical source will be replaced by quantum radiation, the field fluctuations will be directly extracted from the measured variation in interference contrast^30^. Our method, thus, opens the way to the detection of non-classical states of the electromagnetic field, such as Fock or squeezed states^30^.

## Sample design and characterization of single-electron pulses

Our sample (Fig. 1b) is a two-dimensional electron gas (GaAs/AlGaAs) of density *n*_s_ = 1.2 × 10^15^ m^−2^ and mobility *μ* = 1.8 × 10^6^ cm^2^ V^−1^ s^−1^, set at filling factor *ν* = 3 by applying a perpendicular magnetic field *B* = 1.37 T (Supplementary Section E.7). All the measurements were performed at 25 mK—the base temperature of the fridge. The interferometer is defined by two quantum point contacts (QPCs) (Fig. 1b, yellow) used to partition the outer channel at *ν* = 3 with a transmission probability *T*_*i*_ (*i* = A, B) and reflection probability *R*_*i*_ = 1 − *T*_*i*_ (Fig. 1a). This implies that single-electron interferometry occurs on the outer channel of *ν* = 3, whereas the two inner channels (not shown in Fig. 1a) are fully reflected within the cavity.

The red gate in Fig. 1b is used as a plunger to tune the interference pattern. This can be done by applying a d.c. voltage ${V}\,_{\rm{G}}^{{{\rm{d.c.}}}}$ or a time-dependent one ${V}\,_{\rm{G}}^{{{\rm{a.c.}}}}(t)$, either of which modulates the area of the cavity and, thus, the Aharonov–Bohm phase. Ohmic contacts (Fig. 1b, purple) are used for the generation of a periodic train of short single-electron pulses by applying a Lorentzian-shaped^3,7,33,34^ voltage drive *V*_pulse_(*t*), as well as for the measurement of the transmitted d.c. current *I*_out_ and its fluctuations *S*. Both ${V}\,_{\rm{G}}^{{{\rm{a.c.}}}}(t)$ and *V*_pulse_(*t*) are generated by an arbitrary waveform generator (AWG) with a time resolution of 15.6 ps and at a frequency *f* = 1 GHz. As shown in Fig. 1c, we label *τ*_e_ as the temporal width of the single-electron pulse *V*_pulse_(*t*) (*τ*_e_ is approximately a few tens of picoseconds) and ${\tau }_{{\rm{s}}}=\frac{1}{2f}=500\,{\rm{ps}}$ the temporal width of the square voltage ${V}\,_{\rm{G}}^{{{\rm{a.c.}}}}(t)$.

Before studying single-electron interferometry, we first characterize the emitted single-electron excitations. *V*_pulse_(*t*) is a periodic train of Lorentzian voltage pulses, parameterized by the charge carried by each pulse ($qe=\int_{0}^{1/f}\frac{{e}^{2}}{h}{V}_{{{\rm{pulse}}}}(t){{\rm{d}}}t$) and by the temporal width of the pulses *τ*_e_: ${V}_{{{\rm{pulse}}}}(t)={\sum }_{n}\frac{qh{\tau }_{{\rm{e}}}}{\uppi e}\frac{1}{{(t-n/f\,)}^{2}+{\tau }_{{\rm{e}}}^{2}}$. *q* and *τ*_e_ are calibrated by measuring the noise *S* generated by the partitioning of the current *I*_in_(*t*) = *V*_pulse_(*t*) × *e*^2^/*h* at QPC A (whereas QPC B is fully open, *T*_B_ = 1; Fig. 1d). *q* is extracted from the measurement of *S* as a function of both amplitude of the excitation drive generated by the AWG and the temporal width *τ*_e_ of the pulses (Supplementary Section E.2). *τ*_e_ is calibrated by performing Hong–Ou–Mandel (HOM) interferometry^7,10,35^ at QPC A. Two identical trains of single-electron pulses are generated at both inputs of QPC A with a tunable time delay *τ* between the two inputs (Fig. 1d). For large time delays (∣*τ*∣ ≫ *τ*_e_), single-electron excitations generated at the two inputs are independently partitioned, and the noise equals the classical random partition noise Δ*S*_class_ (where Δ*S* refers to the excess noise with respect to equilibrium). By contrast, for short time delays ∣*τ*∣ ≤ *τ*_e_, fermionic antibunching at QPC A suppresses the output noise and Δ*S* decreases close to 0.

Figure 1e presents the measurement of the normalized noise Δ*S*(*τ*)/Δ*S*_class_ for two different widths of pulses generated by the AWG: *τ*_AWG_ = 15.6 ps (blue points) and *τ*_AWG_ = 46.9 ps (orange points). The measured HOM dips are then fitted with a Lorentzian shape, providing an in situ measurement of the width of the emitted pulses: *τ*_e_ = 35 ± 2 ps (blue points) and *τ*_e_ = 63 ± 3 ps (orange points). The increase in the measured width *τ*_e_ compared with *τ*_AWG_ can be explained by the dispersion of the applied voltage pulse when propagating from the AWG to the sample. Figure 1f gathers our measurements of the width *τ*_e_ for the generated pulses of increasing width *τ*_AWG_ (the red points correspond to *q* = 1 pulses and the purple points, to *q* = 2). We observe that the widening of pulses is well captured by an offset of 20 ps of *τ*_e_ with respect to *τ*_AWG_.

## Single-electron interferometry

**Fig. 2:**
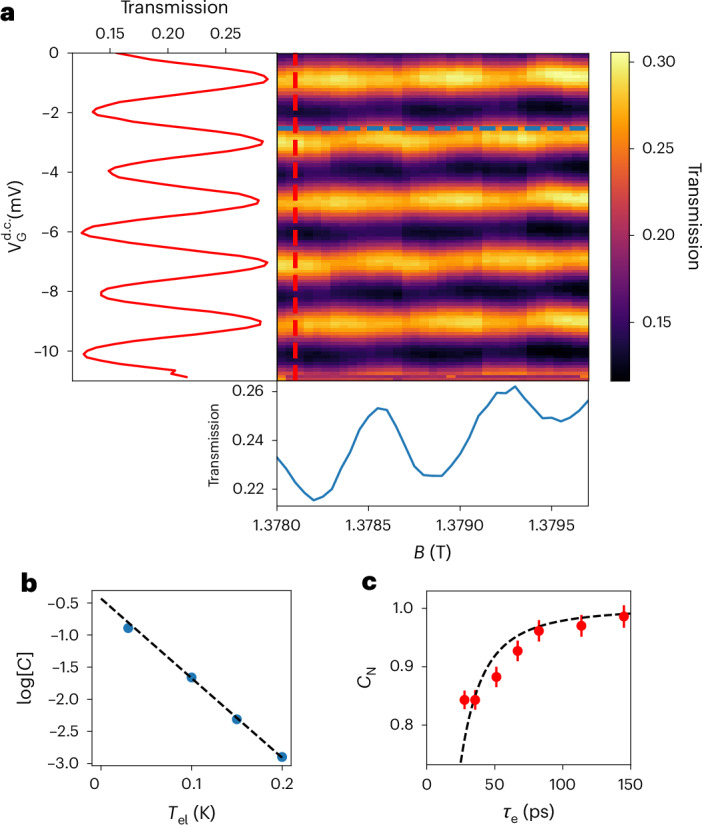
Fabry–Pérot interferometry with single electrons. **a**, Transmission of the FPI as a function of *B* and ${V}_{\rm{G}}^{{{\rm{d.c.}}}}$, showing a periodic behaviour. The two insets show cuts along the dotted lines drawn on the central plot as a function of the field (blue) and d.c. gate voltage (red). **b**, Temperature dependence of the contrast of FPI oscillations. **c**, Evolution of the normalized contrast of oscillations *C*_N_ as a function of the width *τ*_e_ of single-electron Lorentzian pulses. The data points are extracted from a sinusoidal fit of the oscillations of $T({V}\,_{\rm{G}}^{{{\rm{d.c.}}}})$ and the error bars are obtained from the resulting covariance matrix. The dashed line represents the overlap of two Lorentzian wavefunctions *C*_N_(*τ*_L_/*τ*_e_) as a function of *τ*_e_, with *τ*_L_ = 30 ps.

We now move to the measurement of single-electron interferences through the FPI by partitioning the outer channel at both QPC A and QPC B. Figure 2a represents the two-dimensional colour plot of the transmission probability $T({V}\,_{\rm{G}}^{{{\rm{d.c.}}}},B)$ of single-electron excitations through the cavity as a function of ${V}\,_{\rm{G}}^{{{\rm{d.c.}}}}$ and *B*. We observe large oscillations of *T* when varying ${V}\,_{\rm{G}}^{{{\rm{d.c.}}}}$, with a period $\Delta {V}\,_{\rm{G}}^{{{\rm{d.c.}}}}=2.05\pm 0.36\,{\rm{mV}}$ and a peak-to-peak amplitude Δ*T* = 0.15 ± 0.003 corresponding to an interference contrast *C* = Δ*T*/(2〈*T*〉) = 0.35 ± 0.01. The oscillatory pattern is well reproduced by a sinusoidal fit (Supplementary Section E.5). This shows that $T({V}\,_{\rm{G}}^{{{\rm{d.c.}}}},B)$ results from the interference of a single electron with itself after performing one round trip inside the FPI. We also observe oscillations of *T* when varying *B* with a period Δ*B* = 0.63 ± 0.08 mT. This periodicity corresponds to a variation of 2π in the Aharonov–Bohm phase acquired within an area of *h*/(*e*Δ*B*) = 6.6 ± 0.8 μm^2^, which matches well with the area *A* of the FPI. However, the amplitude of the *B* oscillations are roughly five times smaller than the measured oscillations as a function of ${V}\,_{\rm{G}}^{{{\rm{d.c.}}}}$. This can be explained by the effect of the Coulomb interaction within the interferometer^36–38^ (Supplementary Section G). In the following, we focus on the measurement of the interference pattern as a function of ${V}\,_{\rm{G}}^{{{\rm{d.c.}}}}$.

To further characterize the FPI, we measure (Fig. 2b) the evolution of the interference contrast *C* as a function of temperature *T*_el_. The decay of the contrast is well reproduced by an exponential decay with a characteristic temperature scale of ${T}\,_{{{\rm{el}}}}^{0}=81\,{\rm{mK}}$. Considering a thermal averaging of the interference pattern^39^, ${T}\,_{{{\rm{el}}}}^{0}=\hslash /(\uppi {k}_{{\rm{B}}}{\tau }_{{\rm{L}}})$ is related to the time *τ*_L_ = *L*/*v* (where *v* is the electron velocity) it takes for an electron to make one round trip in the cavity. This allows us to estimate *τ*_L_ = 30 ps corresponding to a velocity *v* = 3.8 × 10^5^ m s^−1^.

For short single-electron pulses, the finite travel time inside the cavity *τ*_L_ leads to a reduced overlap of the electronic wavefunction at the interferometer’s output, leading to a reduced interference contrast. For Lorentzian wavepackets of wavefunction ${\varphi }_{{\tau }_{{\rm{e}}}}(t)=\frac{\sqrt{{\tau }_{{\rm{e}}}/\uppi }}{t-{\rm{i}}{\tau }_{{\rm{e}}}}$, the overlap is given by ${C}_{{\rm{N}}}({\tau }_{{\rm{L}}}/{\tau }_{{\rm{e}}})=\Re [\int\,{\rm{d}}t{\varphi }_{{\tau }_{{\rm{e}}}}(t){\varphi }_{{\tau }_{{\rm{e}}}}^{* }(t+{\tau }_{{\rm{L}}})]=\frac{1}{1+{({\tau }_{{\rm{L}}}/2{\tau }_{{\rm{e}}})}^{2}}$. *C*_N_(*τ*_L_/*τ*_e_) is also the interference contrast normalized by its value for *τ*_L_ = 0, that is, *C*_N_(*τ*_L_/*τ*_e_) = *C*(*τ*_L_/*τ*_e_)/*C*(*τ*_L_/*τ*_e_ = 0). Figure 2c represents the evolution of *C*_N_(*τ*_L_/*τ*_e_) as a function of *τ*_e_. In the limit 2*τ*_e_ > *τ*_L_, a small reduction in *C*_N_ is expected. We indeed observe such a small reduction that can be accounted for by the above expression of *C*_N_(*τ*_L_/*τ*_e_) using *τ*_L_ = 30 ps (Supplementary Section E.6).

## Detection of fast electric fields

We now describe our main realization that comprises the detection of fast electric fields using single-electron interferometry. We detect the change in the interference current resulting from the time-dependent voltage ${V}\,_{\rm{G}}^{{{\rm{a.c.}}}}(t)$ applied on the plunger gate (Fig. 1a). We choose a square-shaped voltage of temporal width *τ*_s_ = 500 ps and repetition frequency *f* = 1 GHz. We vary the peak-to-peak amplitude of the generated square from *V*_AWG_ = 75 mV to *V*_AWG_ = 280 mV. *V*_AWG_ is the peak-to-peak amplitude generated at room temperature. After being attenuated at each stage of the fridge, it corresponds to a peak-to-peak amplitude ${V}\,_{\rm{G}}^{{{\rm{a.c.}}}}$ at the level of the plunger gate that varies from 350 μV (for *V*_AWG_ = 75 mV) to 1.3 mV (for *V*_AWG_ = 280 mV). ${V}\,_{\rm{G}}^{{{\rm{a.c.}}}}(t)$ is then detected by measuring the interference pattern $T({V}\,_{\rm{G}}^{{{\rm{d.c.}}}},{t}_{0})$ as a function of both ${V}\,_{\rm{G}}^{{{\rm{d.c.}}}}$ and *t*_0_ the time delay between ${V}\,_{\rm{G}}^{{{\rm{a.c.}}}}(t)$ and *V*_pulse_(*t*) (Fig. 1c).

**Fig. 3:**
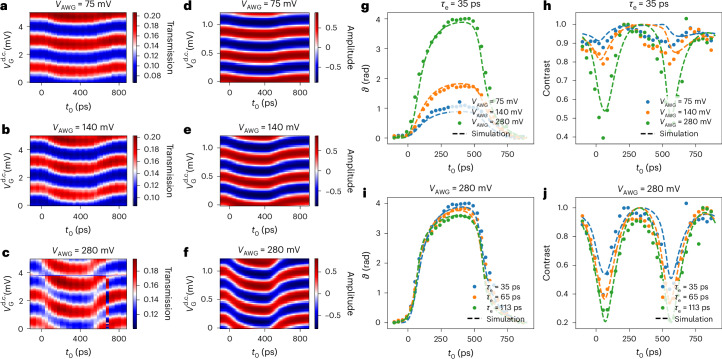
Sensing of a time-dependent voltage with single-electron interferometry. **a**–**c**, Transmission measured at the output of the FPI as a function of delay *t*_0_ between the single-electron pulses *V*_pulse_(*t*) and the square excitation ${V}_{\rm{G}}^{{{\rm{a.c.}}}}(t)$. The three maps show data for Lorentzian pulses of width 35 ps with varying amplitudes of the square excitation *V*_AWG_ = 75 mV, 140 mV and 280 mV. **d**–**f**, Simulations performed using the same parameters as in **a**–**c** (**d**–**f**, respectively), and the rise time of the square voltage is 140 ps. **g**, Evolution of the phase *ϑ* of the oscillations as a function of *t*_0_ for a fixed width *τ*_e_ = 35 ps of the single-electron pulse varying the amplitude *V*_AWG_ of the square drive. **h**, Associated contrast *C*(*t*_0_). **i**, Evolution of *ϑ* of oscillations as a function of *t*_0_ for three widths *τ*_e_ of the single-electron pulses. **j**, Associated contrasts *C*(*t*_0_). The full datasets used to obtain these curves are provided in Supplementary Section E.3. The simulation are shown as dashed lines in **g** and **h**.

Figure 3a–c represents the two-dimensional colour plot of $T({V}\,_{\rm{G}}^{{{\rm{d.c.}}}},{t}_{0})$ for three different amplitudes of the square-voltage excitation. The temporal width of the emitted single-electron pulses is *τ*_e_ = 35 ps. The observed effect of ${V}\,_{\rm{G}}^{{{\rm{a.c.}}}}(t)$ on $T({V}\,_{\rm{G}}^{{{\rm{d.c.}}}},{t}_{0})$ can be easily understood. ${V}\,_{\rm{G}}^{{{\rm{a.c.}}}}(t)$ leads to a phase shift in the interference pattern for *t*_0_ ≈ 100 ps and *t*_0_ = 600 ps corresponding to the time at which the emission of a single electron is synchronized with the sudden variations in the square-voltage excitation. As expected, the measured phase shift increases when the amplitude of the square voltage varies from 75 mV to 280 mV. To extract the temporal variation in ${V}\,_{\rm{G}}^{{{\rm{a.c.}}}}(t)$, for each time delay *t*_0_, we measure the complex contrast $C({t}_{0}){{\rm{e}}}^{{\rm{i}}\vartheta ({t}_{0})}$ of the interference pattern from a sinusoidal fit of $T({V}\,_{\rm{G}}^{{{\rm{d.c.}}}})$ (Supplementary Section E.5). For each amplitude *V*_AWG_, we choose a phase reference $\vartheta ({t}_{0}^{{{\rm{ref}}}})=0$ taken at the first data point at ${t}_{0}^{{{\rm{ref}}}}=-100\,{\rm{ps}}$.

Figure 3g represents our measurement of *ϑ*(*t*_0_) for the three amplitudes of square excitation *V*_AWG_. The shape of the square excitation is well reproduced. As detailed below, for such short electronic wavepackets with *τ*_e_ = 35 ps, the temporal resolution of our voltage measurement is mainly limited by the rise time of 140 ps of the applied square excitation ${V}\,_{\rm{G}}^{{{\rm{a.c.}}}}(t)$ and not by our experimental detection method. Importantly, we observe that the measured phase shift *ϑ*(*t*_0_) scales linearly with the excitation amplitude up to error bars. This implies that our method directly reconstructs ${V}\,_{\rm{G}}^{{{\rm{a.c.}}}}(t)$ from the measurement of *ϑ*(*t*_0_), with ${V}_{{\rm{G}}}^{{{\rm{a.c.}}}}({t}_{0})=\frac{e}{{C}_{{\rm{G}}}}\frac{\vartheta ({t}_{0})}{2\uppi }$, where *C*_G_ = 0.08 ± 0.01 fF is deduced from the d.c. plunger-gate voltage periodicity $\Delta {V}\,_{\rm{G}}^{{{\rm{d.c.}}}}$: ${C}_{{\rm{G}}}=e/\Delta {V}\,_{\rm{G}}^{{{\rm{d.c.}}}}$. This linear relation between the interference phase and detected voltage is important for the accurate reconstructions of voltages in a large dynamical range as well as for future applications of quantum signals. Our voltage resolution is ~50 μV, taken as three times the error bar of the reconstructed phase signal. Note that the sensitivity could be increased by a factor of ten by increasing the size of the plunger gate accordingly, thereby increasing the gate capacitance and reaching the few-microvolt sensitivity. However, this would also slightly decrease the detection time resolution by increasing the coupling time between the gate and single-electronic wavepackets.

The quantum nature of our detection process is illustrated in Fig. 3h, presenting the contrast of the interference *C*(*t*_0_) extracted from the sinusoidal fit mentioned above. To compare the different amplitudes of the detected square-voltage excitation *V*_AWG_, we plot (Fig. 3h) the contrast normalized by the maximal value it reaches when varying *t*_0_. For all the traces, a clear suppression in the contrast is observed for *t*_0_ ≈ 80 ps and 580 ps, corresponding to the times for which ${V}\,_{\rm{G}}^{{{\rm{a.c.}}}}({t}_{0})$ rises up and falls down. Close to these two values of *t*_0_, the different temporal components of the interfering electronic wavepacket (with a characteristic width *τ*_e_) experience different values of the interference phase (corresponding to different values of the square plunger-gate voltage). This leads to a reduction in the interference contrast. As observed in Fig. 3h, the contrast reduction is more pronounced when *V*_AWG_ increases, which leads to an increased spreading of the phase acquired by the different components of the electronic wavepacket. This contrast reduction strikingly demonstrates the quantum nature of the detection process: the interference contrast is reduced by the quantum fluctuations of the position within the single-electronic wavepacket.

The role of the temporal width of the emitted wavepackets is illustrated in Fig. 3i,j, representing *ϑ*(*t*_0_) and *C*(*t*_0_) for a fixed amplitude *V*_AWG_ = 280 mV and different wavepacket widths *τ*_e_ of 35 ps, 65 ps and 113 ps. Figure 3i shows the importance of using short wavepackets for better time resolution in the reconstruction of ${V}\,_{\rm{G}}^{{{\rm{a.c.}}}}(t)$. As observed in Fig. 3i, the extracted temporal evolution of *ϑ*(*t*_0_) is smoothed when increasing *τ*_e_, reducing the amplitude of variation in *ϑ*(*t*_0_) and increasing its rise time. As shown in Fig. 3j, increasing the spread of the electronic wavepacket also enhances the reduction in contrast by quantum fluctuations of the electron position. The contrast dips get increasingly more pronounced when increasing *τ*_e_ from 35 ps to 113 ps.

Our measurements can be well reproduced using a simple model of single-electron interference introduced in ref. ^30^ (Supplementary Sections B and C). We compute the complex contrast of interference $C({t}_{0}){{\rm{e}}}^{{\rm{i}}\vartheta ({t}_{0})}$ in the presence of the time-dependent modulation ${V}\,_{\rm{G}}^{{{\rm{a.c.}}}}(t)$ normalized by its value for ${V}\,_{\rm{G}}^{{{\rm{a.c.}}}}(t)=0$:

1 $$C({t}_{0}){{\rm{e}}}^{{\rm{i}}\vartheta ({t}_{0})}=\frac{\int\,{{\rm{d}}}t{{\rm{e}}}^{{\rm{i}}2\uppi \frac{{C}_{{\rm{G}}}}{e}{V}_{{\rm{G}}}^{{{\rm{a.c.}}}}(t)}{\varphi }_{{\tau }_{{\rm{e}}}}^{* }({t}_{0}-t){\varphi }_{{\tau }_{{\rm{e}}}}({t}_{0}-t-{\tau }_{{\rm{L}}})}{\int\,{{\rm{d}}}t{\varphi }_{{\tau }_{{\rm{e}}}}^{* }(t){\varphi }_{{\tau }_{{\rm{e}}}}(t-{\tau }_{{\rm{L}}})}.$$

The numerator of equation (1) describes the interference between two electronic paths. In the first case, the electron exits the interferometer after propagating through its lower arm only. In the second case, the electron exits the interferometer after performing one round trip of duration *τ*_L_ in the cavity and accumulating the dynamical phase $2\uppi \frac{{C}_{{\rm{G}}}}{e}{V}\,_{{\rm{G}}}^{{{\rm{a.c.}}}}(t)$ when passing below the gate (Supplementary Section B.4). The denominator represents the normalization of contrast by the same interference term in the absence of a dynamical phase (${V}\,_{\rm{G}}^{{{\rm{a.c.}}}}=0$).

The model (Fig. 3g–j, dashed lines) effectively reproduces all the experimental observations, such as the evolution of phase *ϑ*(*t*_0_), its smoothing when increasing the width of the emitted wavepackets *τ*_e_ and the decrease in contrast *C*(*t*_0_) due to quantum fluctuations of the electron position. The agreement between data and model demonstrates our ability to probe time-dependent voltages by exploiting the quantum phase of a single-electron wavefunction.

## Conclusions

We have demonstrated that single-electron quantum states could be used as a fast and sensitive probe of time-dependent voltages. By measuring the phase *ϑ*(*t*_0_) of a single-electron interference pattern in an FPI, we reconstruct a time-dependent voltage ${V}_{G}^{\rm{a.c.}}({t}_{0})$ applied to a metallic gate coupled to one arm of the interferometer. We reach a time resolution of a few tens of picoseconds, limited by the temporal width *τ*_e_ of the emitted wavepackets, and a voltage resolution of a few tens of microvolts. The voltage resolution could be improved by increasing the size of the metallic gate that couples the probed electromagnetic field to the interferometer or by increasing the emission frequency of single electrons, ultimately reaching microvolt resolution. The measurement of the contrast *C*(*t*_0_) demonstrates the quantum nature of our detection method. We observe a sharp decrease in *C*(*t*_0_) close to fast variations in *ϑ*(*t*_0_) caused by the quantum fluctuations of the electron position.

The results presented here concern the detection of classical voltages. Our method can be extended to exotic quantum states of the electromagnetic field, such as Fock or squeezed states generated on chip^40,41^. In the latter case, measuring the enhancement and reduction in the contrast *C* when varying *t*_0_ would directly reflect the enhancement or reduction in the phase fluctuations associated with the squeezing of the electromagnetic field^30^. Finally, the use of the phase of a single-electron wavefunction as the basic element of a quantum detection scheme opens the way for completely new detection methods. For example, it has been proposed^30^ to engineer the electron phase using chirping methods for the detection of terahertz electromagnetic fields. This opens a new and challenging road for the engineering of single-electron states for quantum sensing applications.

## Methods

### Current noise measurements

The current noise at the output of QPC A in the HOM configuration is converted into a voltage noise via the quantum Hall edge channel resistance *R*_*ν*_ = *h*/*νe*^2^ between the output ohmic contact and the ground, as done elsewhere^14^. To move the noise measurement frequency in the megahertz range to avoid parasitic noise contribution at a low frequency, the output ohmic contact is also connected to the ground via an LC tank circuit with a resonance frequency *f*_0_ = 1.1 MHz. The tank circuit is followed by a custom-built cryogenic amplifier and a room-temperature amplifier. A vector signal analyser measures the autocorrelation of the output-voltage noise in a 100-kHz bandwidth centred at *f*_0_. The current noise measurements are calibrated by measuring the thermal noise of the output resistance *R*_*ν*_ as a function of temperature.

### Average current measurements

The d.c. contribution of the output current *I*_out_ generated by the periodic train of single-electron pulses *V*_pulse_(*t*) is measured by a lock-in amplifier by applying a square modulation to *V*_pulse_(*t*). The modulation is performed at 1 MHz, thereby averaging over many pulses generated with a 1-GHz frequency, alternating sign at 1 MHz. *I*_out_ is converted into a voltage signal on the output impedance of the sample, *Z*, which consists of the Hall resistance *R*_*ν*_ in parallel with the LC tank circuit described above. It is then amplified with a total gain *G* by a custom-built cryogenic amplifier followed by a commercial room-temperature amplifier. The current *I*_out_ and charge *qe* carried by each pulse are calibrated by following a procedure described in Supplementary Section E.2.

### Calibration of single-electron pulses

The charge *qe* carried by each pulse is determined by measuring both calibrated excess current noise Δ*S* generated by the partitioning of the single-electron pulses by QPC A and the uncalibrated d.c. input current *I*_in_. We plot on the same graph (Supplementary Section E.2) the noise measurements $\frac{\Delta S}{{T}_{{\rm{A}}}(1-{T}_{{\rm{A}}})}$ obtained for different amplitudes and different widths *τ*_e_ of the generated voltage pulses *V*_pulse_(*t*). These noise measurements are plotted as a function of our measurements of the uncalibrated amplified d.c. input current *G*∣*Z*∣*I*_in_. Remarkably, all the data points fall on a linear slope, reflecting that the noise is proportional to the input current: $\frac{\Delta S}{{T}_{{\rm{A}}}(1-{T}_{{\rm{A}}})}=2e{I}_{{{\rm{in}}}}=2{e}^{2}fq$, where *f* = 1 GHz is the repetition frequency. We can, thus, calibrate the lever arm *α* relating our measurement of the input current to the charge *q*: *α* = *G*∣*Z*∣*I*_in_/*q*. By choosing *α* = 1.81 × 10^−4^ V, we impose that our data $\frac{\Delta S}{{T}_{{\rm{A}}}(1-{T}_{{\rm{A}}})}$ have the expected slope 2*e*^2^*f* when plotted as a function of *q* = *G*∣*Z*∣*I*_in_/*α*. This provides a calibration of both charge per pulse *q* and input current *I*_in_. We can check the soundness of our calibration procedure by plotting the noise $\frac{\Delta S}{{T}_{{\rm{A}}}(1-{T}_{{\rm{A}}})}$ generated by a d.c. voltage bias *V*_d.c._, with *q*_d.c._ = *eV*_d.c._/(*hf*). As shown in Supplementary Section E.2, all our measurements fall appropriately on the expected slope for shot noise: $\frac{\Delta S}{{T}_{{\rm{A}}}(1-{T}_{{\rm{A}}})}=2{e}^{2}fq$.

### Phase and contrast of single-electron interferometric signal

To extract the phase and contrast of the single-electron interferometric signal, we perform cuts on the two-dimensional maps $T({V}\,_{\rm{G}}^{{{\rm{d.c.}}}},{t}_{0})$ at fixed *t*_0_. The transmission *T* is extracted from the measurement of the d.c. current *I*_out_ as *T* = *I*_out_/*I*_in_, where *I*_in_ is the d.c. contribution of *I*_in_(*t*): *I*_in_ = *qef*. The cuts show an oscillating signal as a function of ${V}\,_{\rm{G}}^{{{\rm{d.c.}}}}$ (Fig. 2a), which is fitted using a cosine function of the form $C({t}_{0})\cos ({V}\,_{\rm{G}}^{{{\rm{d.c.}}}}/{V}_{0}+\vartheta ({t}_{0}))+b$. The fit parameter *ϑ*(*t*_0_) is then used to plot the data shown in Fig. 3g,i. The contrast plotted in Fig. 3h,j is then calculated as the ratio *C*(*t*_0_)/max(*C*(*t*_0_)). From these fits, we observe that there is no second-harmonic contribution to the signal and that a simple sinusoidal oscillation describes our experimental data perfectly, justifying the use of a model in which a single round trip inside the FPI cavity is taken into account.

To prevent any correlation between one duty cycle and the next one, the duration of the pulse and the time to make one round trip in the cavity are short compared with 1/*f*.

## Online content

Any methods, additional references, Nature Portfolio reporting summaries, source data, extended data, supplementary information, acknowledgements, peer review information; details of author contributions and competing interests; and statements of data and code availability are available at 10.1038/s41565-025-01888-2.

## Supplementary information

Supplementary Information Supplementary Sections A–G and Figs. 1–16.

## Data Availability

All data plotted in the Article or its Supplementary Information are available via Zenodo (10.5281/zenodo.13768874)^42^. Other data that support the findings of this study are available from the corresponding authors upon reasonable request.
