# Supplementary material for: Time-resolved sensing of electromagnetic fields with single-electron interferometry
Source: Nat Nanotechnol. 2025 Mar 17;20(5):596–601. doi: 10.1038/s41565-025-01888-2 (PMC12095073; doi:10.1038/s41565-025-01888-2)
Supplement: Supplementary file 1 — Supplementary Sections A–G and Figs. 1–16. [file 41565_2025_1888_MOESM1_ESM.pdf]

nature nanotechnology

Supplementary information https://doi.org/10.1038/s41565-025-01888-2

Time-resolved sensing of electromagnetic fields with single-electron interferometry

In the format provided by the authors and unedited

CONTENTS

A. Figure of merit of the detection 1

B. Presentation of the setup and modelization 2
1. Fabry-Perot configuration 2
2. HOM configuration 3
3. Modeling strategies 3
4. Main results 4

C. Computing the interference contrast 5
1. Phase of the interferometer ϕ_{AB} 5
2. Free electron discussion 6
a. General form of the result 6
b. Propagation along branch 1 6
c. Ballistic propagation 7
3. Electronic decoherence within each branch 8
a. Modeling interactions via EMP scattering 8
b. Results 8
c. Adiabatic approximation 9
4. Coupling between the two branches 10

D. Filtering of $\mathcal{F}_{V_G}(t)$ 11
1. Long wavepackets 11
2. Short wavepackets 11

E. Measurement setup and processes 11
1. Fridge setup 11
2. Pulse calibration 12
3. Full experimental data set 14
4. Square drive calibration 14
5. Analysis procedure 15
6. Contrast vs. amplitude of gate oscillations 16
7. Characterization of the QPCs 17

F. Numerical modelization 17
1. Modelization of the square drive 17
2. Continuous evaluation of the parameters 17

G. Study of the interference pattern in the Fourier space and effects of the Coulomb interaction 19

References 19

Appendix A: Figure of merit of the detection

Let us consider zero-point fluctuations in circuit QED like systems (see Fig. 1.A). The charge operator can be written as

$$\hat{Q} = -iQ_{\mathrm{ZPF}}(\hat{a} - \hat{a}^{\dagger}), \tag{A1}$$

where $Q_{\mathrm{ZPF}} = \sqrt{\hbar/2Z}$, with Z the characteristic impedance of the line (see Fig. 1.A), typically equal to $50\,\Omega$. Taking the mean value of the square of the charge leads to $\langle \hat{Q}^2 \rangle_{|N\rangle} \simeq Q^2_{\mathrm{ZPF}}(2N+1)$ where N is the mean number of photons in our circuit. The charge Q is linked to the voltage V through the capacitance C such that $V = Q/C$, which allows us to write the voltage fluctuations in the N photon Fock state $|N\rangle$ of the LC-resonator:

$$\langle V^2 \rangle_{|N\rangle} = \left(\frac{Q_{\mathrm{ZPF}}}{C}\right)^2 (2N+1). \tag{A2}$$

The approximate voltage increase $\Delta V_{1\mathrm{ph}}$ associated to the presence of a single photon ($N = 1$) in the circuit is $\Delta V_{1\mathrm{ph}} \simeq \sqrt{3}\frac{Q_{\mathrm{ZPF}}}{C}$. Rewriting Q_{ZPF} as $e\sqrt{R_K/4\pi Z}$ where R_K is the quantum of resistance h/e^2 leads to $\Delta V_{1\mathrm{ph}} \simeq \sqrt{\frac{\hbar}{Z}}\frac{1}{C}$. The characteristic impedance of the line can be written in terms of the line inductance and capacitance as $Z = \sqrt{L/C}$, and so can the frequency $2\pi f = 1/\sqrt{LC}$. Combining those quantities we have

$$e\Delta V_{1\mathrm{ph}} = \frac{e^2}{C}\sqrt{\frac{\hbar}{Ze^2}} = \frac{e^2}{C}\sqrt{\frac{R_K}{2\pi Z}} = \frac{e^2}{C}\frac{1}{\sqrt{2\pi z}} = \frac{e^2}{C}\frac{hf}{\sqrt{2\pi z}}\frac{\sqrt{LC}}{\hbar} = hf\sqrt{2\pi z}., \tag{A3}$$

where z is the ratio of the characteristic impedance of the line and the quantum of resistance Z/R_K.

Assuming a $50\,\Omega$ characteristic impedance of the line we have in our experiment $z = 2\times 10^{-3}$. Applying this result to our measurement at $f = 10\,\mathrm{GHz}$ we obtain an equivalent voltage associated to the presence of a single photon $\Delta V_{1\mathrm{ph}} \simeq 4\,\mu\mathrm{V}$. Therefore, with an estimated experimental voltage resolution of $50\,\mu\mathrm{V}$ our current apparatus has an equivalent detection resolution of about 10 photons. This number could be greatly improved by modifying the geometric parameters of the driving gate.

Appendix B: Presentation of the setup and modelization

1. Fabry-Perot configuration

The Fabry-Perot (FP) interferometer set-up is presented in Fig. 2A where single electrons are injected from the source S. The two QPCs behave as ideal electronic beam splitters. A perpendicular magnetic field is applied, generating an Aharonov-Bohm phase ϕ_{AB} which, in full generality, is related to the magnetic flux Φ_B enclosed between the arms 1 and 2 as well as on the dc-component of the plunger gate voltage V_G^{dc} (see Ref. [1] and a discussion specific to the device considered here in Sec. C 4). The classical time-dependent voltage $V_G(t)$ in the orange box is probed through the measurement of the average current at the detector D which reads

$$i_D(t) = \langle \hat{\psi}_D^\dagger(t)\hat{\psi}_D(t)\rangle_S = -e\Big(I_0(t) + 2\,\mathrm{Re}\big[\mathrm{e}^{-\mathrm{i}\phi_{AB}}I_+(t)\big]\Big) \tag{B1}$$

with $\hat{\psi}_D(t)$ being the fermionic operator at the detector at time t and $I_+(t)$ being the interference contribution to the average electrical current. Here $\langle\ldots\rangle_S$ denotes an average taken over the many body state corresponding to the source S switched on.

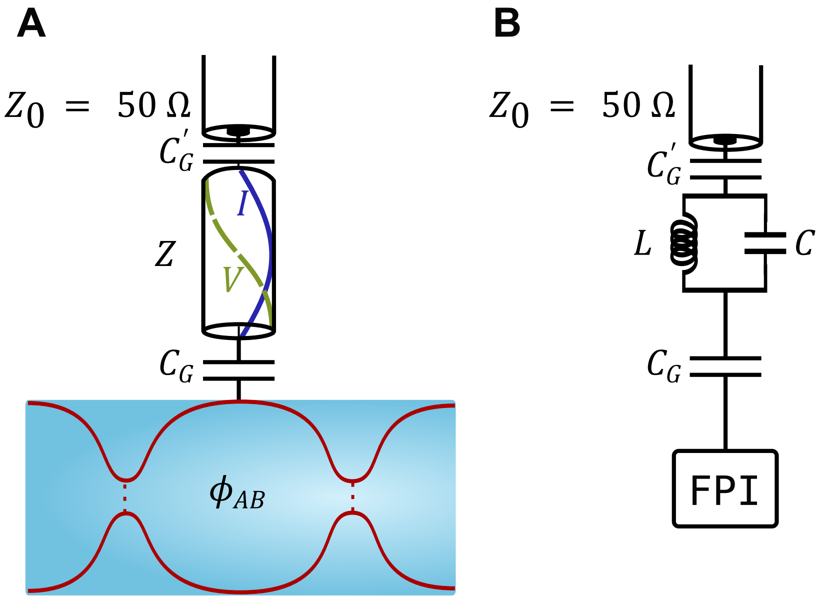

FIG. 1. **Estimating the detector figure of merit: (A)** We consider an rf resonator containing one photon. The ground state is associated to a given current I and voltage V that couples to the FPI through the capacitance C_G. **(B)** This configuration can be seen as an LC resonator of capacitance C and inductance L capacitively coupled to the FP.

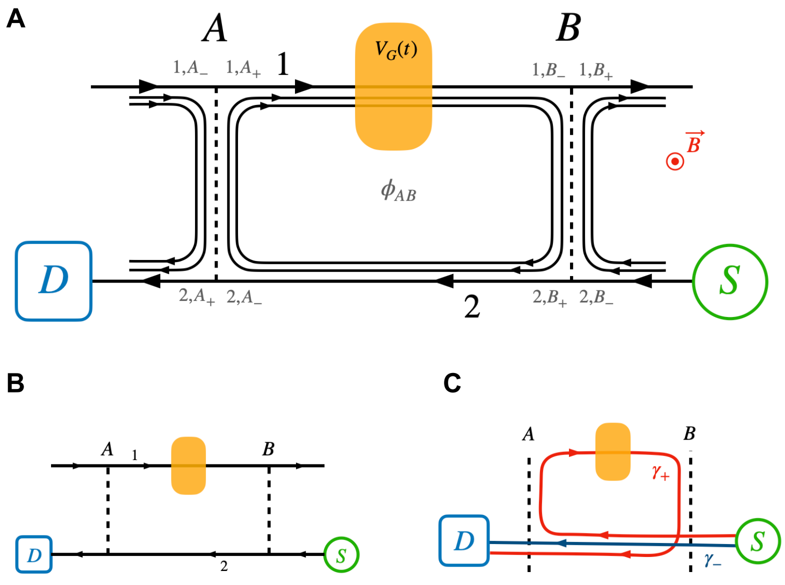

FIG. 2. **(A)** Full schematic of the Fabry-Perot interferometer at $\nu = 3$. The QPCs A and B operate in the weak-backscattering (WB) regime where the transport channel is the outer one. The orange box in the upper branch is the plunger gate $V_G(t)$ we want to probe by measuring the interference contribution to the average dc current at the detector D. **(B)** The Fabry-Perot interferometer in the weak backs-cattering regime with only charge transporting channel represented. **(C)** Visualization of the interference paths $\gamma_\pm$ from the source S to the detection D in this configuration.

Since the interferometer is operating in the weak-backscattering (WB) limit (i.e. $R_\alpha \ll 1$) we expect the dominant paths to the interference to be the ones in Fig. 2C: the single electron can either go straight through the two quantum point contacts (QPCs), reaching the detector (blue path γ_-), or turn around the inner cavity making one lap and a half (red path γ_+).

2. HOM configuration

The device is also calibrated in a configuration based on Hong-Ou-Mandel (HOM) interferometry as depicted on Fig. 3A. In this mode, QPC B is fully open and HOM calibration is performed at QPC A and used to identify Lorentzian voltage pulses there. An important point is that in the calibration modes, the inner channels appearing on Fig. 2A are no longer closed.

This implies that, in this mode, all the channels of branch 2 are excited symmetrically by S. Since S consists of exciting the charge mode of branch 2, it means that all edge channels of branch 2 carry the same coherent state of edge-magnetoplasmons (EMPs). In the HOM configuration (see fig.1D-F of main text), HOM interferometry is performed on the outer edge channel and therefore it can be viewed as a characterization of the EMP coherent state within each of these edge channels.

It is important to notice that, in this operation mode, the electromagnetic environment of the outer edge channel of branch 2 is not the same than in the FP configuration since the geometry of all the other channels is closed.

3. Modeling strategies

A first difficulty comes from the fact that the calibration mode does not characterize the excitations arriving at QPC A in the FP configuration. In principle, since we are dealing with coherent EMP states, it is possible to infer the coherent state of EMPs injected by S into branch 2 and then to discuss the FP configuration.

The method used to discuss the FP configuration consists of back-propagating the fermionic field $\hat{\psi}_D(t)$ along the two relevant interfering paths thereby formulating it in a way similar to Mach-Zehnder (MZ) interferometry [2]. The averages are then computed using the state emitted by S.

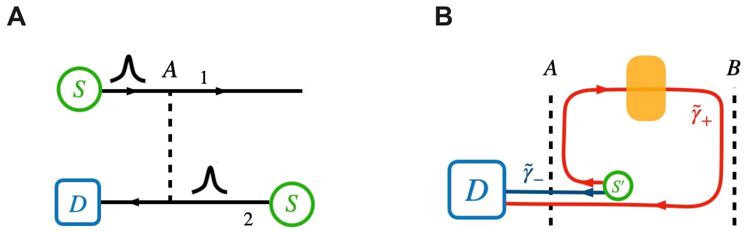

FIG. 3. **(A)** The characterization of Lorentzian pulses is done experimentally at QPC A by HOM interferometry. **(B)** Visualization of the new interference paths $\tilde{\gamma}_\pm$ assuming single electron excitations have been injected right before QPC A.

However, the analogy is not straightforward due to the coupling of the outer channels of the two branches via the closed edge channels. In order to understand the system, we will discuss increasingly complex models of the FP interferometer that incorporate more and more features that have just been discussed.

First of all, in Sec. C 2, we neglect Coulomb interactions and use time dependent single particle scattering to obtain the interference contribution to the dc average current. This simple model, despite its limitations, clearly depicts the analogy with MZ interferometry and enables to see the role of the various time of flights in the system. We derive the contrast of the interferences on the dc average current in terms of the overlap between a Leviton wavepacket delayed by the full time of flight around the Fabry-Perot loop and itself and a filter applied to the time dependent phase imprinted on the electrons by the time dependent plunger gate voltage $V_G(t)$.

Then, Coulomb interactions are introduced in two steps: first, in Sec. C 3, we assume that branches 1 and 2 of the FP interferometer (see Fig. 3B) are not coupled via Coulomb interactions. This accounts for Coulomb interactions within each of them as well as capacitive couplings to environmental degrees of freedom, each branch having its own environment. However, as shown on Fig. 2A, this does not account for the presence of closed edge channels when the device is operated in the FP configuration. Nevertheless, when injecting electronic excitations with energies much below any resonance frequency associated with these closed edge channels, we can expect the uncoupled branches model to be valid. The main result of this analysis is that, at very low temperatures, and provided that the time dependent voltage $V_G(t)$ is slow compared to the characteristic times associated with electronic decoherence, the relative contrast between the interference signal at non zero $V_G(t)$ and at $V_G(t) = 0$ can be computed using free electron expressions derived in Sec. C 2.

In Section C 4, we discuss the case where the two branches are coupled via the closed edge channels and argue qualitatively that the effect related to the closed edge channels may be weak in the domain of operation of the experiment.

Finally, in Sec. D, we study the relation between the relative contrast and the interference signal at non zero $V_G(t)$ and at $V_G(t) = 0$ and the phase imprinted on the electrons by the top gate.

4. Main results

Let us now summarize the main results of the theoretical modelization details in the next sections. The quantity which is measured is the dc average current which depends on the Aharonov-Bohm phase ϕ_{AB} in a 2π-periodic way

$$\langle i_D^{(\mathrm{dc})}\rangle_S \simeq -e\left(I_0^{(\mathrm{dc})} + \mathrm{e}^{-\mathrm{i}\phi_{AB}} I_+^{(\mathrm{dc})} + \mathrm{e}^{\mathrm{i}\phi_{\mathrm{AB}}} I_-^{(\mathrm{dc})}\right) \tag{B2}$$

where we only have retained the first harmonics in ϕ_{AB} in accordance with experimental data. Note that the interference contribution $I_+^{(\mathrm{dc})} = \left(I_-^{(\mathrm{dc})}\right)^*$ depends on the wavepackets injected by S, that is their duration τ_e as well as their injection time t_0 and of course, of the time dependent top gate voltage $V_G(t)$.

We define the relative interference contrast as the ratio of $I_+^{(\mathrm{dc})}$ for a given $V_G(t)$ to the same quantity for $V_G(t) = 0$. It is a complex number that depends on experimentally controlled parameters t_0, τ_e as well as $V_G(t)$. Our theory provides an explicit prediction for this relative contrast valid even in the presence of decoherence effects, provided they manifest themselves only at frequencies much higher than the ones involved in $V_G(t)$:

$$C(t_0)\,\mathrm{e}^{\mathrm{i}\vartheta(t_0)} = \int_{\mathbb{R}} \mathrm{e}^{\mathrm{i}\phi_1(V_G,\tau)} f_{\mathrm{FP}}(t_0-\tau)\mathrm{d}\tau \tag{B3}$$

where $e^{i\phi_1(V_G,\tau)}$ is the phase imprinted on the electrons by the top gate voltage. This expression for the contrast is the general form of eq.1 of the main text. For a short top gate located in the middle of branch 1, and assuming that $V_G(t)$ does not vary during the characteristic time with the plunger gate capacitively coupled to the edge channel, it can be expressed as

$$e^{i\phi_1(V_G,t)} = e^{2\pi i C_G V_G\left(t-\frac{\tau_1}{2}\right)/e} \tag{B4}$$

in which C_G denotes the electrochemical capacitance associated with the capacitor built from the top gate and the quantum Hall edge channel beneath it. Note that the variation in gate voltage δV_G induces a proportional modulation in the area δA [3]. In the CD regime, where we are operating, special attention must be given to the capacitance C_G, as it reflects the capacitance of the entire system, including both the open and closed regions. Assuming we use Leviton electronic excitations of wavefunction[4]

$$\varphi_{\tau_e}(t) = \sqrt{\frac{\tau_e}{\pi}}\,\frac{1}{t - i\tau_e}\,, \tag{B5}$$

the filter function $f_{\mathrm{FP}}(\tau)$ takes the "universal" form:

$$f_{\mathrm{FP}}(\tau) = \frac{\varphi_{\tau_e}(\tau-\tau_L)\,\varphi^*_{\tau_e}(\tau)}{\langle\varphi_{\tau_e}|^{\tau_L}\varphi_{\tau_e}\rangle} \tag{B6}$$

where the overlap

$$\langle\varphi_{\tau_e}|^{\tau_L}\varphi_{\tau_e}\rangle = \int_{\mathbb{R}} \varphi^*_{\tau_e}(t)\varphi_{\tau_e}(t-\tau_L)\,\mathrm{d}t \tag{B7}$$

is the absolute contrast in the absence of interactions within the Fabry-Perot interferometer. To have an observable interference signal, the experiment is performed in a regime where $\tau_e \simeq 35$ to $150\,$ps which is larger than $\tau_L \simeq 30\,$ps: the electronic wavepackets are wider than the Fabry-Perot loop.

The explicit expression of the filter $f_{\mathrm{FP}}(\tau)$ is given by Eq. (C23) and and its behavior in modulus and phase is discussed in details in Sec. D. In the experiment $\tau_L/2\tau_e \lesssim 0.5$, we have shown that $f_{\mathrm{FP}}(\tau)$ probes the electrical phase $e^{\phi_1(V_g,t)}$ over a time window of width $\sim \tau_e$ around t_0 with a smooth phase modulation. This ultimately justifies why the Fabry-Perot interferometer can be seen as a time resolved probe of this phase.

Appendix C: Computing the interference contrast

1. Phase of the interferometer ϕ_{AB}

Before discussing the propagation of the single electronic excitation injected by the source S into the interferometer, let us comment on the phase ϕ_{AB} appearing in Eqs. (B1) and (B2).

This phase corresponds to the static electromagnetic phase associated with the propagation of an electronic destruction operator along a certain closed path γ (see Fig. 2C). In presence of a static electrical potential $V(\mathbf{r})$ and vector potential $\mathbf{A}(\mathbf{r})$, the phase accumulated by a charge $-e$ propagating between times t_i and t_f is

$$\phi[\gamma] = \frac{q}{\hbar}\int_{t_i}^{t_f} \left[\dot{\mathbf{r}}(t)\cdot\mathbf{A}(\mathbf{r}(t)) - V(\mathbf{r}(t))\right]\,\mathrm{d}t \tag{C1a}$$

$$= -2\pi\,\frac{e\Phi_B}{h} + \frac{e}{\hbar}\int_{t_i}^{t_f} V(\mathbf{r}(t))\,\mathrm{d}t \tag{C1b}$$

in which the first term corresponds to the Aharonov-Bohm magnetic phase associated with the magnetic flux Φ_B enclosed by the curved path γ and the second term is the electrostatic phase associated with the static component of the electrical potential experienced by the particle during its motion. In a Fabry-Perot quantum Hall interferometer, this phase depends on the precise geometry of the edge channel along which the fermionic operator $\psi_D(t)$ is propagated, as explained in [1].

When Coulomb interactions are not taken into account, it contains a dependence on the dc voltage applied to the plunger gate V_G^{dc} because the shape of the edge channel, and thus the total area enclosed by γ depends on V_G^{dc}.

When Coulomb interactions are taken into account, the second term in the r.h.s of Eq. (C1b) must be taken into account and reflect static charges present in the system. In the Fabry-Perot configuration, these are the charges

stored in the closed channel appearing on Fig. 2A. These charges are not expected to vary during an experimental run since these channels are closed. Of course, it depends on the capacitive coupling between these channels and the channels 1 and 2 since it determines the phase accumulated by one electron along the path γ_+ shown on Fig. 2C. But the discussion of Ref. [1] could be applied to analyze this phase more quantitatively. The mixed dependence in the external magnetic field B and the dc plunger gate voltage determines the stripped pattern of the interference contrast as a function of these two parameters. As explained in the main body of this work, the interferometer is operated in a regime where Coulomb interactions are quite strong, but not in the extreme Coulomb dominated regime where the B dependence is expected to disappear [1].

This static phase being isolated, the electronic operator $\psi_D(t)$ will be back-propagated along the paths depicted on Fig. 2C or 3B within the various models considered in this section: time dependent single particle scattering first and then, taking into account Coulomb interactions between charged hydro-dynamical modes (EMPs) and other dynamical degrees of freedom.

2. Free electron discussion

We start by considering the FP interferometer operating in a regime where Coulomb interaction effects between electrons can be neglected. They will nevertheless experience the effect of the external time dependent potential imposed by the top gate. This amounts to using a time dependent single particle scattering approach. Under this assumption, we can consider that electrons propagate within one edge channel as in Fig. 2B.

a. *General form of the result*

In the weak-backscattering regime, we evaluate the average interference current by back-propagating the fermionic field along $\gamma_\pm$ in Fig. 2C. When the source injects a single electron excitation in wavefunction φ_S, the interference contribution to the average current at time t is given by:

$$I_+(t) = -T_A T_B \sqrt{R_A R_B} \int_{\mathbb{R}^2} \mathcal{Z}_2^*(t-t_-) \big(\mathcal{Z}_2 * R_1 * \mathcal{Z}_2\big)(t,t_+)\, \varphi_S(t_+)\varphi_S^*(\tau_-)\, \mathrm{d}t_+ \mathrm{d}t_- \tag{C2}$$

where the convolution is defined as

$$\big(\mathcal{Z}_2 * R_1 * \mathcal{Z}_2\big)(t,t') = \int_{\mathbb{R}^2} \mathcal{Z}_2(t_A - t') R_1(t_A, t_B) \mathcal{Z}_2(t - t_B)\, \mathrm{d}t_A \mathrm{d}t_B\,. \tag{C3}$$

in which $\mathcal{Z}_2(\tau)$ denotes the amplitude for propagation from B_+ to A_- along branch 2 in a time τ and $R_1(t,t')$ denotes the single particle scattering amplitude from $(1, A_+)$ to $(1, B_-)$ at respective times t' and t. Note that due to the application of the time dependent potential V_G it does depend on $t - t'$ as well as of $(t + t')/2$. By contrast, $\mathcal{Z}_2$ only depends on time differences since electrons propagating along channel 2 do not experience the time dependent potential $V_G(t)$. The convolution $\big(\mathcal{Z}_2 * R_1 * \mathcal{Z}_2\big)(t,t')$ represents the total single particle scattering amplitude along path γ_+, up to the QPC reflection and transmission amplitudes which have been taken out for convenience.

b. *Propagation along branch* 1

The scattering amplitude $R_1(t,t')$ contains information about the phase acquired by the electrons when experiencing the influence of $V_G(t)$. The geometry of the sample suggests that the electrons only feel $V_G(t)$ beneath the top gate. Since propagation is chiral, the most general expression for $R_1(t,t')$ is

$$R_1(t,t') = \int_{\mathbb{R}^2} R_{1\backslash G,<}(t'_G - t') R_G(t_G, t'_G) R_{1\backslash G,>}(t, t_G) \mathrm{d}t_G\, \mathrm{d}t'_G \tag{C4}$$

in which $R_{1\backslash G,<}(t'_G - t')$ (resp. $R_{1\backslash G,>}(t, t_G)$) represents the amplitude to travel across the part of branch 1 before (resp. after) the parts beneath the top gate between times t' and t'_G (resp. t_G and t). The amplitude $R_G(t_G, t'_G)$ then represents the amplitude for the particle to enter the region beneath the top gate at time t'_G and exit it at time t_G. It depends on the time dependent top gate voltage. If we assume that propagation beneath this short top gate is ballistic with time of flight τ_G and that the electrons feel the time dependent $V_G(t)$, we have

$$R_G(t_G, t'_G) = \delta(t_G - t'_G - \tau_G)\, \mathrm{e}^{\frac{\mathrm{i}e}{\hbar}\int_{t'_G}^{t_G} V_G(\tau)\, \mathrm{d}\tau}\,. \tag{C5}$$

This discussion shows that because of the integration over t_G, the electrical potential felt by the electrons may get blurred by quantum spreading of the wavepacket during its propagation before the top gate. Such an effect would certainly limit the time resolution of the interferometer.

We expect this dispersive blurring to be present whenever the inverse of the duration τ_e of the electronic wavepacket is of the order of the energy scale at which linear dispersion for electrons propagating within the edge channels is not constant. In the integer quantum Hall regime, this is expected to occur for $\tau_e^{-1} \gtrsim \omega_c$, where ω_c is the cyclotron frequency which is usually of the order of the terahertz: $\omega_c/2\pi \sim 0.45\,\mathrm{THz} \times B\,(\text{in T})$ in AlGaAs/GaAs. This may become an issue when single electron wavepackets of duration close or even below 1 ps are used, but not in the present experiment where $\tau_e \geq 30\,\mathrm{ps}$.

c. Ballistic propagation

These considerations suggest that we should here restrict ourselves to ballistic propagation within the FP interferometer. In the case of branch 1, denoting by τ_1 the total time of flight and by τ_G the time of flight beneath the top gate, we have:

$$R_{1\backslash G,<}(\tau) = R_{1\backslash G,>}(\tau) = \delta\left(\tau - \frac{\tau_1 - \tau_G}{2}\right) \tag{C6}$$

and using Eqs. (C4) and (C5), this leads to

$$R_1(t,t') = \mathrm{e}^{\mathrm{i}\phi_1[V_G,t]}\delta(t - t' - \tau_1) \tag{C7}$$

where the phase $\phi_1[V_G,t]$ is

$$\phi_1[V_G,t] = \frac{e}{\hbar}\int_{-\tau_G/2}^{\tau_G/2} V_G\left(t - \frac{\tau_1}{2} + \tau\right)\mathrm{d}\tau \sim \frac{e\tau_g}{\hbar}\,V_G\left(t - \frac{\tau_1}{2}\right) \underset{\tau_G = R_K C_G}{=} 2\pi\frac{C_G V_G(t - \tau_1/2)}{e}\,. \tag{C8}$$

The last approximation is valid in the limit of $\tau_G \ll \tau_1$ and $V_g(t)$ varying slowly over time scales $\sim \tau_G$. Ballistic propagation along branch 2 is described by $\mathcal{Z}_2(\tau) = \delta(\tau - \tau_2)$. When a single electron excitation of wavefunction φ_S is injected by S, the interference contribution to the dc average current is of the form $I_+^{(\mathrm{dc})} = T_A T_B\sqrt{R_A R_B}\,X_+^{(\mathrm{dc})}$ with

$$[X_+^{(\mathrm{dc})}]_{V_G} = \int_{\mathbb{R}} \varphi_S(t - \tau_L)\,\varphi_S^*(t)\,\mathrm{e}^{\mathrm{i}\phi_1(V_G,t-\tau_2)}\mathrm{d}t\,. \tag{C9}$$

in which $\tau_L = \tau_1 + \tau_2$ is the total time of flight along the loop. Let us now specialize this for a Lorentzian excitation of duration τ_e is injected by S at a time t_0:

$$\varphi_S(t) = \varphi_{\tau_e}(t - t_0) \quad \text{with } \varphi_{\tau_e}(t) = \sqrt{\frac{\tau_e}{\pi}}\,\frac{1}{t - \mathrm{i}\tau_e}\,. \tag{C10}$$

This leads to the following expression ($\tau_{12} = \tau_1 - \tau_2$ and $\tau_L = \tau_1 + \tau_2$):

$$[X_+^{(\mathrm{dc})}]_{V_G} = \int_{\mathbb{R}} \frac{\tau_e}{\pi}\,\frac{\mathrm{e}^{\mathrm{i}\phi_1(V_G,t_0-\tau)}}{\left(\tau + \frac{\tau_{12}}{2}\right)^2 + \left(\tau_e - \frac{\mathrm{i}\tau_L}{2}\right)^2}\,\mathrm{d}\tau\,. \tag{C11}$$

In particular, for $V_g = 0$, we find the vacuum baseline

$$[X_+^{(\mathrm{dc})}]_0 = \int_{\mathbb{R}} \varphi_{\tau_e}(t - \tau_L)\,\varphi_{\tau_e}^*(t)\,\mathrm{d}t = \frac{2\tau_e}{2\tau_e - \mathrm{i}\tau_L} \tag{C12}$$

which leads to the following final result

$$\frac{[X_+^{(\mathrm{dc})}]_{V_G}}{[X_+^{(\mathrm{dc})}]_0} = \frac{1}{\pi}\int_{\mathbb{R}} \mathrm{e}^{\mathrm{i}\phi_1(V_G,t_0-\tau)}\,\frac{\tau_e - \frac{\mathrm{i}\tau_L}{2}}{\left(\tau + \frac{\tau_{12}}{2}\right)^2 + \left(\tau_e - \frac{\mathrm{i}\tau_L}{2}\right)^2}\,\mathrm{d}\tau\,. \tag{C13}$$

This expression shows that the relative contrast $[X_+^{(\mathrm{dc})}]_{V_G}/[X_+^{(\mathrm{dc})}]_0$, as a function of t_0 appears as a convolution of the electrical phase $\mathrm{e}^{\mathrm{i}\phi_1(V_G,t)}$ by a kernel that only depends on the geometry of the interferometer (times of flights τ_1 and τ_2) and of the duration τ_e of the Lorentzian pulses. Remarkably, as we will see now, the result of Eq. (C13) is robust to electronic decoherence.

3. Electronic decoherence within each branch

a. *Modeling interactions via EMP scattering*

In this section, propagation of electrons within the two branches of the interferometers is modeled within the bosonization formalism, which assumes that the underlying free theory is based on electrons with a linear dispersion relation. The effect of Coulomb interactions can then be conveniently treated within the edge-magnetoplasmon scattering formalism [5, 6]. Assuming that the electron fluid is in the linear screening regime, the corresponding EMP scattering theory is linear.

In this section, we assume that Coulomb interactions do not couple branches 1 and 2 of the FP interferometer. This amounts to ignoring the effects of the closed edge channels that appear on Fig. 2A but, as will be discussed in Sec. C 4, we expect their effects to be important only for $\omega \gtrsim 2\pi v/L$ where L is the perimeter of the FP loop. The main hypothesis of this section is that the parts of channels 1 and 2 are independent EMP scatterers described by the following EMP scattering amplitudes:

$$b_{1,\mathrm{out}}(\omega) = t_1(\omega) b_{1,\mathrm{in}}(\omega) + r_1(\omega) a_{1\mathrm{in}}(\omega) + \kappa_1(\omega)\, V_G(\omega) \tag{C14a}$$

$$b_{2,\mathrm{out}}(\omega) = t_2(\omega) b_{2,\mathrm{out}}(\omega) + r_2(\omega) a_{2\mathrm{in}}(\omega) \tag{C14b}$$

in which the $a_{1/2}(\omega)$ are the environmental modes associated to the edge channels 1 and 2. There may be one or several of such modes some of which may or may not carry charge but we assume that energy conservation ensures unitarity of the total scattering matrix for these bosonic excitations. The transmission amplitudes are then related to the finite frequency admittance $Y_{11}(\omega)$ and $Y_{22}(\omega)$ via the usual expression [6, 7]:

$$Y_{\alpha\alpha}(\omega) = \frac{e^2}{h}(1 - t_\alpha(\omega)) \tag{C15}$$

whith the usual convention that the current is defined as the total current entering the region of the edge channel under consideration: $I_\alpha = i_{\alpha,\mathrm{in}} - i_{\alpha,\mathrm{out}}$. In a similar way, the coefficient $\kappa_1(\omega)$ describes the frequency dependent linear response of the edge current $i_{11,\mathrm{out}}(\omega)$ to $V_G(\omega)$. More precisely

$$Y_{1,G}(\omega) = e\sqrt{\omega}\,\kappa_1(\omega) \tag{C16}$$

Under the hypothesis of the present subsection, edge channel 2 does not respond to $V_G(t)$ when the QPC are opened and this is why there is no linear term involving $V_G(\omega)$ in Eq. (C14b) contrary to Eq. (C14a).

b. *Results*

As in the previous section the idea is to back-propagate the fermionic field $\hat{\psi}_D(t)$ along the two main paths in order to connect the FP geometry to the MZ formalism in the same spirit as in Ref. [2]. When a single electron excitation with wavefunction φ_S is injected, the average time-dependent current is then obtained as

$$I_+(t) = -T_A\sqrt{R_A R_B}\, \mathrm{e}^{2\mathrm{i}\theta_2} \mathrm{e}^{\mathrm{i}\phi_1(V_G,t)} \int \mathrm{d}t_+ \mathrm{d}t_-\, \mathcal{Z}_2(t_- - (t-\tau_2))\, \mathcal{Z}_1(t-\tau_2-t_+)\, \varphi_S^*(t_-)\varphi_S(t_+) \tag{C17}$$

where the phase $\phi(V_G, t)$ appears as the convolution of the gate voltage by a kernel which describes the filtering associated with the capacitive coupling to the top gate. More specifically, introducing the finite frequency admittance of the dipole formed by the plunger gate and the branch 1 of the FP interferometer:

$$\phi_1(V_G, t) = \frac{e}{\hbar}\left(\Gamma_{1,G} * V_G\right)(t) \tag{C18a}$$

$$\widetilde{\Gamma}_{1,G}(\omega) = \frac{R_K Y_{1,G}(\omega)}{-\mathrm{i}\omega} \tag{C18b}$$

In principle, one should therefore use a model of electrostatics of the system to derive this finite frequency admittance, for example in the spirit of the discrete element modeling of a top gate capacitively coupled to an edge channel in Ref. [2]. But due to the geometry of the sample considered here, one can use the expression obtained in Eq. (C8). Note that the phase

$$\theta_2 = \mathrm{Im}\left[\int_0^{+\infty} \frac{\mathrm{d}\omega}{\omega}\left(t_2^*(\omega)\mathrm{e}^{\mathrm{i}\omega\tau_2} - 1\right)\right]. \tag{C19}$$

does not depend on time. The two amplitudes $\mathcal{Z}_1(\tau)$ and $\mathcal{Z}_2(\tau)$ correspond to elastic scattering amplitude for a single electron excitation on top of the Fermi sea propagating across branches 1 and 2 respectively. As in Ref. [2], they are expressed in terms of the elastic scattering amplitudes for energy resolved single electron excitations $\widetilde{Z}_\alpha(\omega>0)$ via a Fourier transform (see Refs. [8, 9]):

$$\mathcal{Z}_\alpha(\tau)=\int_0^{+\infty}\widetilde{\mathcal{Z}}_\alpha(\omega)\,\mathrm{e}^{-\mathrm{i}\omega\tau}\,\frac{\mathrm{d}\omega}{2\pi} \tag{C20}$$

and therefore contain not only the information about the Wigner-Smith time delay for low frequency excitations but also about electronic decoherence along the branches 1 and 2 of the Fabry-Perot interferometer. Putting all these results together leads to the following expression

$$\left[X_+^{(\mathrm{dc})}\right]_{V_G}=e^{2i\theta_2}\int_{\mathbb{R}^3}\mathrm{e}^{\mathrm{i}\phi_1(V_G,t-\tau_2)}\mathcal{Z}_2(t_--(t-\tau_2))\,\mathcal{Z}_1(t-\tau_2-t_+)\,\varphi_S^*(t_-)\varphi_S(t_+)\mathrm{d}t_+\mathrm{d}t_-\mathrm{d}t \tag{C21}$$

which, for $\varphi_S(t)=\varphi_{\tau_e}(t-t_0)$ in which φ_{τ_e} defined by Eq. (B5), can then be rewritten as a filtering

$$\left[X_+^{(\mathrm{dc})}\right]_{V_G}=\mathrm{e}^{2\mathrm{i}\theta_2}\int_{\mathbb{R}}\widetilde{f}_{\mathrm{FP}}(\Omega)\widetilde{\mathcal{F}}_{V_G}(\Omega)\,\mathrm{e}^{-\mathrm{i}\Omega t_0}\,\frac{\mathrm{d}\Omega}{2\pi} \tag{C22}$$

of $\mathcal{F}_{V_G}(t)=\mathrm{e}^{\mathrm{i}\phi_1(V_g,t)}$ by the filter

$$\widetilde{f}_{\mathrm{FP}}(\Omega)=4\pi\tau_e\int_{|\Omega|/2}^{+\infty}\mathrm{e}^{-2\omega\tau_e}\,\widetilde{\mathcal{Z}}_1\left(\omega-\frac{\Omega}{2}\right)\,\widetilde{\mathcal{Z}}_2\left(\omega+\frac{\Omega}{2}\right)\frac{\mathrm{d}\omega}{2\pi}\,. \tag{C23}$$

Note that here, the Lorentzian shape of the current pulse is responsible for the $\mathrm{e}^{-\omega\tau_e}$ in the r.h.s. of Eq. (C23): it reflects the Leviton's exponentially decaying wavefunction in energy. Finally, the vacuum baseline is given by

$$\left[X_+^{(\mathrm{dc})}\right]_0=\widetilde{f}_{\mathrm{FP}}(\Omega=0)=4\pi\tau_e\int_0^{+\infty}\mathrm{e}^{-2\omega\tau_e}\,\widetilde{\mathcal{Z}}_1(\omega)\,\widetilde{\mathcal{Z}}_2(\omega)\frac{\mathrm{d}\omega}{2\pi} \tag{C24}$$

Note that, as expected, this expression as the same form as in Ref. [2] with $\mathcal{Z}_1(\omega)\mathcal{Z}_2(\omega)$ playing the role of $\mathcal{Z}_1(\omega)$.

c. Adiabatic approximation

It is then convenient to isolate the contribution of the Wigner-Smith time delays $\tau_{1,2}$ for the EMP modes respectively propagating along branches 1 and 2 of the FPI, by rewriting

$$\widetilde{\mathcal{Z}}_\alpha(\omega)=\mathrm{e}^{\mathrm{i}\omega\tau_\alpha}\widetilde{\mathcal{Z}}_\alpha^{(0)}(\omega) \tag{C25}$$

in which $\widetilde{\mathcal{Z}}_\alpha^{(0)}(\omega)$ contains all the effects of decoherence associated with the dispersion and scattering of the EMP modes ($\widetilde{\mathcal{Z}}_\alpha^{(0)}(\omega)=1$ for ballistic propagation with time of flight τ_α). The filter function $f_{\mathrm{FP}}(\Omega)$ can then be rewritten as

$$\widetilde{f}_{\mathrm{FP}}(\Omega)=4\pi\tau_e\mathrm{e}^{-|\Omega|\tau_e}\mathrm{e}^{\mathrm{i}(|\Omega|\tau_L-\Omega\tau_{12})}\int_0^{+\infty}\mathrm{e}^{-2\omega\tau_e}\mathrm{e}^{\mathrm{i}\omega\tau_L}\,\widetilde{\mathcal{Z}}_1^{(0)}\left(\omega+\frac{|\Omega|-\Omega}{2}\right)\,\widetilde{\mathcal{Z}}_2^{(0)}\left(\omega+\frac{|\Omega|+\Omega}{2}\right)\frac{\mathrm{d}\omega}{2\pi}\,. \tag{C26}$$

Exactly as in Ref. [2], we perform an adiabatic approximation by assuming that, whenever the frequencies involved in $\mathcal{F}_{V_G}(t)$ are much lower than the frequencies at which the amplitudes $\widetilde{\mathcal{Z}}_\alpha^{(0)}$ vary, then the Ω dependance in their argument in the r.h.s. of Eq. (C26) can be neglected. This leads to

$$\widetilde{f}_{\mathrm{FP}}(\Omega)=e^{-|\Omega|\tau_e}e^{i(|\Omega|\tau_L-\Omega\tau_{12})/2}\left[X_+^{(\mathrm{dc})}\right]_0\,. \tag{C27}$$

Substituting this expression in the filtering equation (C22) enables us to obtain the relative contrast as a convolution:

$$\frac{[X_+^{(\mathrm{dc})}]_{V_G}}{[X_+^{(\mathrm{dc})}]_0}=\int_{\mathbb{R}}f_{\mathrm{FP}}(\tau)\,\mathcal{F}_{V_G}(t_0-\tau)\,\mathrm{d}\tau \tag{C28}$$

in which the convolution kernel is obtained as

$$f_{\mathrm{FP}}(\tau)=\frac{1}{\pi}\,\frac{\tau_e-\frac{\mathrm{i}\tau_L}{2}}{\left(t+\frac{\tau_{12}}{2}\right)^2+\left(\tau_e-\frac{\mathrm{i}\tau_L}{2}\right)^2} \tag{C29}$$

This is exactly the same expression as in Eq. (C13). This proves that, in the absence of coupling between the two branches and when the adiabatic approximation is valid, the filtering of the time dependent voltage is identical to the one obtained via time dependent single particle scattering (see Sec. C 2), up to renormalization of the vacuum baseline due to electronic decoherence.

Such a result could indeed be expected since the adiabatic approximation means that information about the time dependent phase $\mathcal{F}_{V_G}(t)$ is stored into edge-magnetoplasmon modes that are not too much affected by dispersion and dissipation: their transmission amplitudes across branches 1 and 2 are close to the ballistic amplitudes $\mathrm{e}^{\mathrm{i}\omega\tau_{1,2}}$. This explains why electronic decoherence is unaffected by the presence of the time dependent voltage $V_G(t)$. Note however that the theory presented here enables, in principle, to account for non-adiabatic effects by using Eq. (C26) instead of Eq. (C27).

4. Coupling between the two branches

In the previous section, Coulomb interactions have been introduced under the hypothesis that the upper and lower branches 1 and 2 of the FP interferometer are not coupled electrostatically. However, due to the presence of closed edge channels on Fig. 2A, this is not true. Here, we discuss the effects of Coulomb interactions in the presence of such closed loops. Let us stress that the effect of the total (static) charge stored on the closed channels has already been incorporated in the effective Aharonov-Bohm phase and therefore, we are only discussing the effect of Coulomb interactions on the so-called hydrodynamic (EMP) modes.

This leads to a more complex scattering matrix for the edge-magnetoplasmon modes of the upper branch $\hat{b}_1$ and of the lower one $\hat{b}_2$. We have to introduce scattering amplitudes $t_{12}(\omega)$ and $t_{21}(\omega)$ respectively connecting $\hat{b}_{1,\mathrm{out}}(\omega)$ to $\hat{b}_{2,\mathrm{in}}(\omega)$ and $\hat{b}_{2,\mathrm{out}}(\omega)$ to $\hat{b}_{1,\mathrm{in}}(\omega)$. These EMP transmission coefficients are related to the finite frequency admittances of the edge channels when the QPCs are fully opened via

$$Y_{\alpha\beta}(\omega)=\frac{e^2}{h}\left(1-t_{\alpha\beta}(\omega)\right)\,. \tag{C30}$$

Then, the $\hat{b}_{2,\mathrm{out}}(\omega)$ modes now responds to the gate voltage $V_G(\omega)$ through a coefficient $\kappa_2(\omega)$ associated with the finite frequency admittance $Y_{2,G}(\omega)$. Finally, exactly as before, there may be other environmental modes to ensure charge conservation as well as energy conservation so that the full scattering matrix for all these bosonic degrees of freedom is unitary.

A crude estimate can be used at low frequencies to obtain an order of magnitude of the finite frequency admittance between the two branches 1 and 2 of the FP interferometer. First of all, folding each of these branches together enables us to see them as a transmission line of characteristic impedance $R_K/2$. They are capacitively coupled to a closed loop which forms a quantum Hall Fabry Perot interferometer (see Ref. [10] for a similar but slightly different geometry) for EMP modes propagating associated with the $\nu_{\mathrm{FP}}-1=2$ inner channels. At low frequency, such an interferometer can be roughtly seen as the series addition of two capacitances and an RL circuit where $R=R_K/2(\nu_{\mathrm{FP}}-1)$ and $L=2R\,\tau_{RL}$ where τ_{RL} is the characteristic RL time associated with a quantum Hall bar [11] at filling fraction $\nu_{\mathrm{FP}}-1$ whose length is of the order of the cavity's perimeter (up to geometric factors). In the end, the finite frequency admittance of such a dipole is of the order

$$R_KY(\omega)=-\mathrm{i}(\nu_{\mathrm{FP}}-1)\,\frac{\omega}{\omega_{\mathrm{LC}}}+\mathcal{O}\left(\frac{\omega}{\omega_{\mathrm{LC}}}\right)^2 \tag{C31}$$

in which $\omega_{\mathrm{LC}}=1/\sqrt{LC}$ is the resonance frequency of the LC resonator which, here, corresponds to the lowest resonance frequency of the EMP cavity formed by the $\nu_{\mathrm{FP}}-1$ closed edge channels of the FP interferometer. This explains why, at low frequencies compared to this first resonance frequency, the coupling between the edge channel 1 and 2 is indeed small.

Of course, it would be interesting to account for the effect of this capacitive coupling mediated by the closed edge channels (see Ref. [12] in the stationary regime) but we think that the experimental data clearly show that it is not relevant for the experiment presented in this paper.

Appendix D: Filtering of $\mathcal{F}_{V_G}(t)$

Let us now discuss the time resolution associated with Leviton wavepackets of duration τ_e which follows from the filter $f_{\mathrm{FP}}(\tau)$ whose expression is, for $\tau_1 = \tau_2$ (as in the sample under consideration):

$$f_{\mathrm{FP}}(\tau) = \frac{1}{\pi}\,\frac{\tau_e - \frac{\mathrm{i}\tau_L}{2}}{\tau^2 + \left(\tau_e - \frac{\mathrm{i}\tau_L}{2}\right)^2}\,. \tag{D1}$$

Analyzing the modulus of this function as a function of τ shows that there are two distinct regimes: the first one which corresponds to *long wavepackets* ($\tau_e < \tau_L/2$) and the regime of *short wavepackets* ($\tau_e > \tau_L/2$).

The regime of short wavepackets is the regime of interest in the present experiment since, in order to observe a significant contrast even for $V_G(t) = 0$, one has to chose $\tau_L \lesssim \tau_e$ since, for example, when interactions can be neglected, the vacuum baseline whose modulus gives the contrast of interference fringes reduces to:

$$\left[X_+^{(\mathrm{dc})}\right]_0 = \frac{\tau_e}{\tau_e - \frac{\mathrm{i}\tau_L}{2}}\,. \tag{D2}$$

1. Long wavepackets

In this regime, $|f_{\mathrm{FP}}(\tau)|$ has its maximum for $\tau = 0$ and then decays to zero for $|\tau|/\tau_e \gg 1$. As shown on Fig. 4A, as soon as $\tau_e \gtrsim \tau_L$, the filter function has a width of the order of τ_e. The study of its phase, depicted on Fig. 4B, shows that when $\tau_e \gg \tau_L/2$, it is almost constant over the interval over which the modulus takes significant values (typically $|\tau/\tau_e| \lesssim 2$). This shows that, in this limit, the filter function $f_{\mathrm{FP}}(\tau)$ tends to "average" the phase factor $\mathcal{F}_{V_G}(t)$ over a window of width τ_e around t_0 in Eq. (B3).

For the values considered in the present work, this is not exactly the case, especially when considering the shortest wavepackets. Nevertheless, the model considered here enables us to account quantitatively of the deviation from the simple image of a simple averaging of the phase.

2. Short wavepackets

When considering short wavepackets ($\tau_e < \tau_L/2$), the behavior of $f_{\mathrm{FP}}(\tau)$ changes drastically. First of all the modulus $|f_{\mathrm{FP}}(\tau)|$ has two maxima for $\tau \simeq \pm\tau_L/2$ which are associated with peaks of width $\sim \tau_e$ as can be seen on Fig. 4C. The phase of $f_{\mathrm{FP}}(\tau)$ is also different: as depicted on Fig. 4D, it starts from $-\arctan(\tau_L/2\tau_e)$ before the two peaks ($-\infty < \tau < -\tau_L/2$) and then switches to $\arctan(\tau_L/2\tau_e)$ between the peaks and goes back to the previous negative value when τ increases above $\tau_L/2$. The transition happens over a duration $\sim \tau_e$.

The appearance of the phase kink for short electronic wavepackets on Fig. 4D is reminiscent of Ref. [13] where the effect of the phase jump induced by a Lorentzian pulse of duration much shorter than the time of flight across a coherent electronic interferometer (FP or MZI) is discussed. Nevertheless, Ref. [13] discusses the influence of the many-body phase jump which is $2\pi\overline{n}$ for a current pulse of charge $-e\bar{n}$ (see Eq. (B4) here). In particular, it discusses the behavior as a function of $\bar{n}$. By contrast, in the present work, $\bar{n} = 1$ and, since we are in the IQH regime, we are discussing the effect of the phase imprinted on the single electronic excitation on top of the Fermi sea. Its wavefunction also exhibits a phase jump of the same shape but with amplitude π (not 2π, see Eq. (B5)). Considering the a many-body phase is indeed important for probing many-body effects and in particular, discussing the role of anyonic statistics of fractionally charged current pulses in the IQH regime [14, 15]. But in the present work, this is not what we are interested in: because we are using one single electron excitation as a probe of the time dependent voltage, we are considering the effects of the time dependent phase and modulus of the Leviton electronic excitation as a function of the ratio its duration τ_e to τ_L.

Appendix E: Measurement setup and processes

1. Fridge setup

All the measurements presented in this paper were performed at the bottom of a dilution cryostat at base electronic temperature 30 mK. The rf lines used to send the pulse and square drive to the sample are represented in figure 5. Both rf lines are attenuated throughout the descent to low temperature by a total of -52 dB. The gate line is

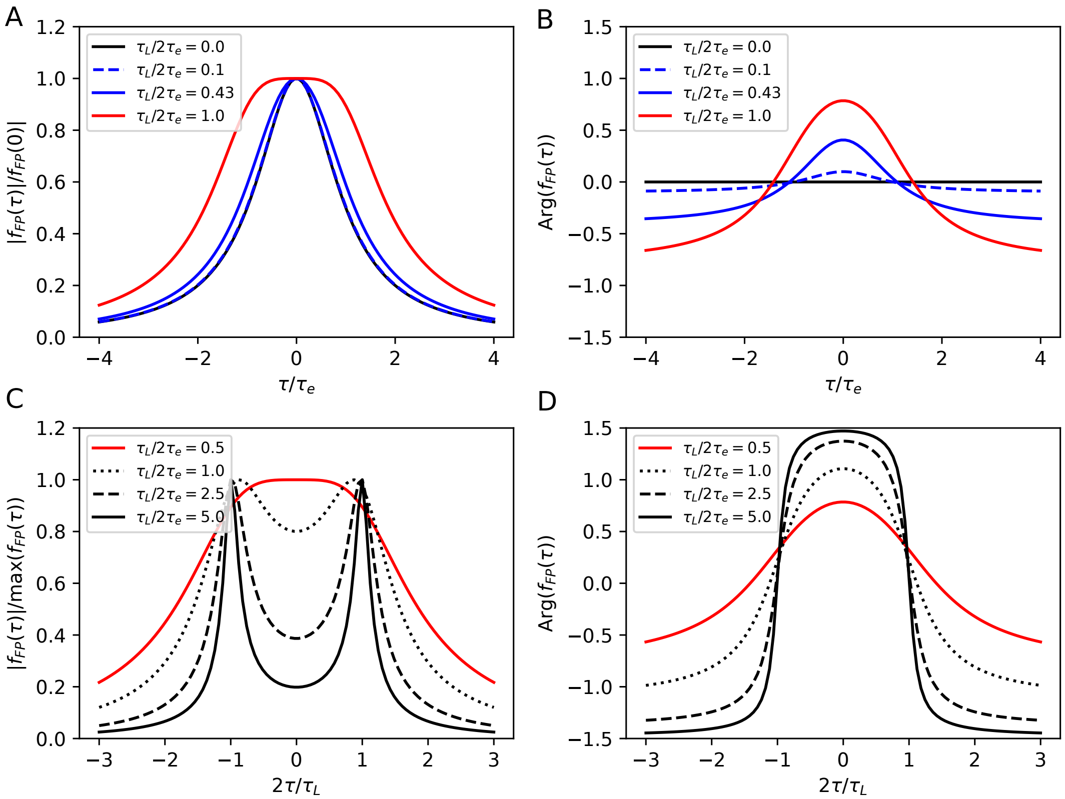

FIG. 4. **(A) and (B)**: plot of $|f_{\rm FP}(\tau)/f_{\rm FP}(0)|$ and $\mathrm{Arg}(f_{\rm FP}(\tau))$ as a function of τ/τ_e for long wave packets: $\tau_L/2\tau_e \to 0$ (grey line), $\tau_L/2\tau_e = 1/10$ (blue dashed line), 0.43 (blue line), 1 (red line). The blue curves correspond to the experimentally realized τ_e ranging from 35 ps to 150 ps with $\tau_L = 30$ ps. **(C) and (D)**: Plot of $|f_{\rm FP}(\tau)|/\max_\tau(|f_{\rm FP}(\tau)|)$ and of $\mathrm{Arg}(f_{\rm FP}(\tau))$ as a function of $2\tau/\tau_L$ for short wavepackets: $\tau_L/2\tau_e = 1$ (red line), 2 (black dotted line), 5 (black dashed line) and 10 (black line).

also connected in dc via a bias tee placed at the level of the mixing chamber. Additionally, a voltage divider is placed outside the fridge on the dc part of the gate. The gate line is capacitively coupled to the sample through the capacitance C_G.

The output signal for the measurement of noise ΔS or output current $I_{\rm out}$ passes through a tank circuit and is collected by a cryogenic amplifier before being amplified at room temperature. The tank circuit is a band-pass filter for the measurement of the noise at a frequency of 1.1 MHz in a 100 kHz bandwidth. It prevents the noise measurement to be polluted by unavoidable low frequency parasitic contributions.

The output current $I_{\rm out}$ is measured by a lock-in amplifier by applying a square modulation to the voltage excitation $V_{\rm pulse}(t)$ used to generate the periodic train of single electron pulses. The modulation is performed at 1 MHz, thus averaging over many pulses generated with a 1 GHz frequency, alternating sign at 1 MHz.

2. Pulse calibration

In our experiment, single electron pulses are generated by applying a periodic train of Lorentzian pulses $V_{\rm pulse}(t)$ containing 1 electron. More generally, a train of Lorentzian pulses containing the charge q (in units of the electron charge) and of temporal width τ_e can be written as $V_{\rm pulse}(t) = \sum_n \frac{qh\tau_e}{\pi e} \frac{1}{(t-n/f)^2+\tau_e^2}$. $V_{\rm pulse}(t)$ is generated at room temperature by an arbitrary wave generator (AWG) generating the time-dependent voltage :

$$V_{\rm pulse,AWG}(t) = \sum_n V_{\rm exc} \frac{\tau_{\rm samp}^2}{(t-n/f)^2 + \tau_{AWG}^2}, \tag{E1}$$

where $\tau_{\rm samp} = 15.6$ ps is the sampling time of the AWG. $V_{\rm pulse,AWG}(t)$ is then attenuated at each stage of the fridge, requiring a proper calibration between the applied voltage at room temperature characterized by $V_{\rm exc}$ and $\tau_{\rm AWG}$ and the charge per pulse q. The calibration of q is performed together with the calibration of the input dc current $I_{\rm in}$,

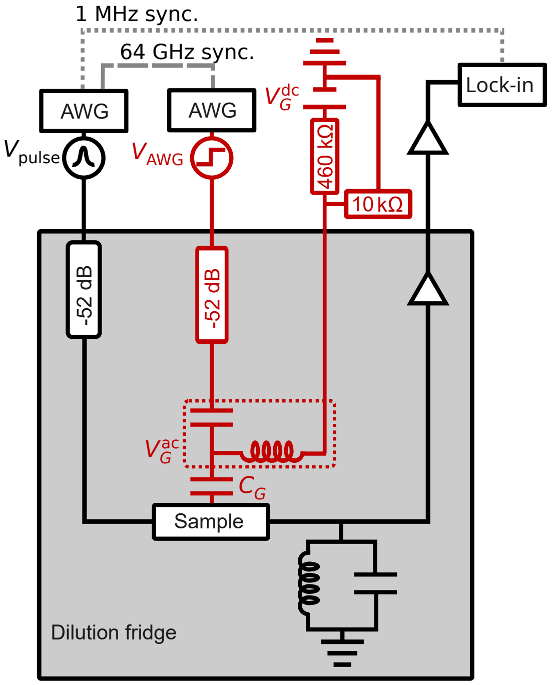

FIG. 5. **Fridge setup:** The sample is placed at the bottom of a dilution refrigerator. It is connected to two AWG outputs through attenuators distributed over the various stages of the fridge for an total attenuation of $-52\,\mathrm{dB}$. The line connected to the gate is also connected in dc via a bias-tee where a voltage divider is present. The output signal is filtered by a tank circuit and then amplified at two stages and acquired by a lock-in amplifier.

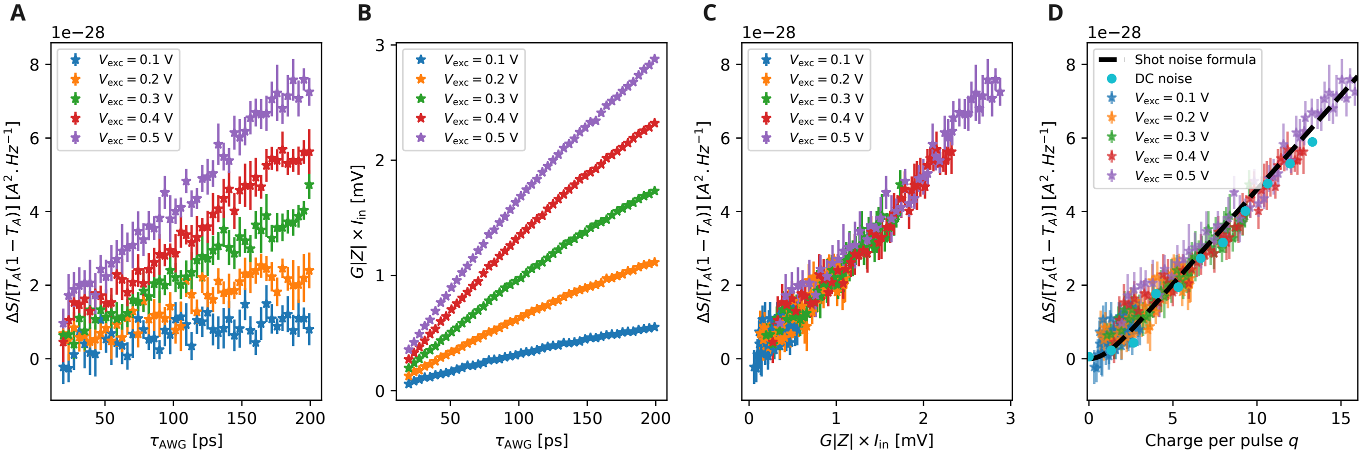

FIG. 6. **Pulse calibration:** (**A**) Shot noise measured in the configuration presented in figure 1.D of the main text as a function of the width τ_{AWG} of the pulses for various amplitudes V_{exc} of the voltage pulse generated by our AWG. (**B**) Measurement of $G|Z|I_{\mathrm{in}}$ as a function of the width τ_{AWG} and the excitation amplitude V_{exc} (**C**) Same data as in A and B, plotting the noise ΔS as a function of $G|Z|I_{\mathrm{in}}$. (**D**) Same data as in C, rescaling $G|Z|I_{\mathrm{in}}$ by the factor $\alpha = 1.81 \cdot 10^{-4}$ V, such that the horizontal axis now represents the total charge contained within a single pulse, $q = G|Z|I_{\mathrm{in}}/\alpha$. The dashed line represents the expected behavior for shot noise and the blue dots the DC noise.

where I_{in} is the dc component of $e^2/hV_{\mathrm{pulse}}(t)$. I_{in} is converted to a voltage on the output impedance Z of the sample that consists in the Hall resistance in parallel with the LC tank circuit used for the noise measurements. It is then amplified by cryogenic and room temperature amplifiers (see Fig.5) with total gain G. It is measured by a lock-in amplifier by applying a square modulation of $V_{\mathrm{pulse,AWG}}(t)$ at the low frequency of 1 MHz. The whole measurement process of I_{in} (modulation, conversion to a voltage on the frequency dependent impedance $Z(\omega)$, gain of the amplifiers) requires a proper calibration.

This is performed by measuring both the calibrated excess current noise ΔS generated by the partitioning of the

train of pulses at QPC1 and the uncalibrated dc current I_{in} for different values of the parameters V_{exc} and τ_{AWG}. The noise and current are related via the Fano factor F defined as the ratio of the noise over the Poissonian limit, $\Delta S = 2eFT_A I_{\text{in}}$. In the limit of a single channel conductor, one has $F = 1 - T_A$ [16], reflecting the binomial process for single electron scattering by QPC A with probability T_A. We thus plot on Figs.6A and B our measurements of the excess noise ΔS normalized by the binomial factor $T_A(1-T_A)$ and of the amplified current $G|Z| \times I_{\text{in}}$. Both the noise and the current show a linear increase for $\tau_{\text{AWG}} \leq 100$ ps. This is expected as in the limit of non-overlapping pulses, q is expected to depend linearly on both τ_{AWG} or V_{exc}. For larger values of τ_{AWG}, a sublinear variation of $\frac{\Delta S}{T_A(1-T_A)}$ and $G|Z|I_{\text{in}}$ is observed that reflects the overlap of consecutive pulses in the train.

We plot on Figs.6C the noise $\frac{\Delta S}{T_A(1-T_A)}$ as a function of $G|Z|I_{\text{in}}$. Remarkably, all points fall on a linear slope. This reflects that the shot noise is proportional to the input current : $\frac{\Delta S}{T_A(1-T_A)} = 2eI_{\text{in}} = 2e^2 fq$, where $f = 1$ GHz is the repetition frequency. We can thus calibrate the lever arm α relating our measurement of the input current to the charge q, $\alpha = G|Z|I_{\text{in}}/q$. By choosing $\alpha = 1.81 \cdot 10^{-4}$ V, we impose that our data ΔS have the expected slope $2e^2 f$ when plotted as a function of $q = G|Z|I_{\text{in}}/\alpha$ (see Fig.6D). This provides both a calibration of the charge per pulse q and of the input current I_{in}.

As a check of the soundness of our calibration procedure, we also plot on Fig.6D the noise ΔS generated by a dc voltage bias V_{dc}, with $q_{\text{dc}} = eV_{\text{dc}}/(hf)$. As can be seen on the figure, all our measurements fall nicely on the expected slope for shot noise $\Delta S = 2e^2 fq$. The dashed line represents the shot noise formula taking into account the temperature: $\Delta S = 2e^2 fq \times \left[\coth\left(\frac{hfq}{2k_B T_{\text{el}}} - \frac{2k_B T_{\text{el}}}{hfq}\right)\right]$ and using the temperature $T_{\text{el}} = 25$ mK.

3. Full experimental data set

In the main text of the article we only present three maps of the measurement of $T(V_G^{\text{dc}}, t_0)$. However more data was used to extract the points in figures 3I and J from the main text. The full data set used for these figures as well as additional data points are shown in figure 7.

In particular, figure 7K was obtained by applying a rectangular drive (with a temporal width $\tau_s = 250$ ps) instead of a square one on $V_G^{\text{ac}}(t)$. As a result, we observe that the transmission changes on a shorter time scale. Figure 8 presents the extracted phase and amplitude of the transmission extracted from figure 7J and K. On this figure the dots represent the experimental data while the dashed lines represent the simulation performed using the same parameters as in the main text, but adapting it to the physical parameters used to measure figures 7J and K. It should be noted here that the amplitude of the rectangle is larger (350 mV) that for the square (280 mV). However, we observe that the phase shift (as seen in figure 8A) does not change in the same proportions, probably due to the rise time that becomes comparable with the temporal width of the rectangular drive ($\tau_s = 250$ ps). The measured variations of the phase are well reproduced by the model both for the square (blue dashed line, $\tau_s = \frac{1}{2f}$) and rectangle (orange dashed line, $\tau_s = \frac{1}{4f}$) drives. While the quantitative agreement is not as good for the contrast as for the phase, we still reproduce the contrast dips in the correct position.

4. Square drive calibration

In order to calibrate the amplitude of the square drive V_G^{ac}, we study the transmission of the FPI in the dc regime, i.e. where instead of sending a pulse we only apply a dc voltage on the input of the interferometer. In the meantime, we change the amplitude of the square drive V_G^{ac} for periodic signals at 1 GHz (figure 9A) and 100 MHz (figure 9B). We observe that, for the right value of the ac amplitude of the square signal V_G^{ac}, the oscillations of the transmission vanish and transmission becomes flat as a function of V_G^{dc}. This specific amplitude of the square drive corresponds to a π phase shift, such that the interference pattern is averaged out between the phases 0 and π. The amplitude of the square voltage for which this happens changes based on the frequency of the square drive, at 1 GHz the π shift happens at $V_G^{\text{ac}} = 280$ mV, and at 100 MHz, this shift happens at $V_G^{\text{ac}} = 220$ mV. This discrepancy originates from an attenuation at 1 GHz that is not present at 100 MHz. At higher amplitude of the square drive, the contrast reappears with a π phase shift of the oscillations. In figure 9B, we observe some jumps that can be attributed to random charge effects within the resonator.

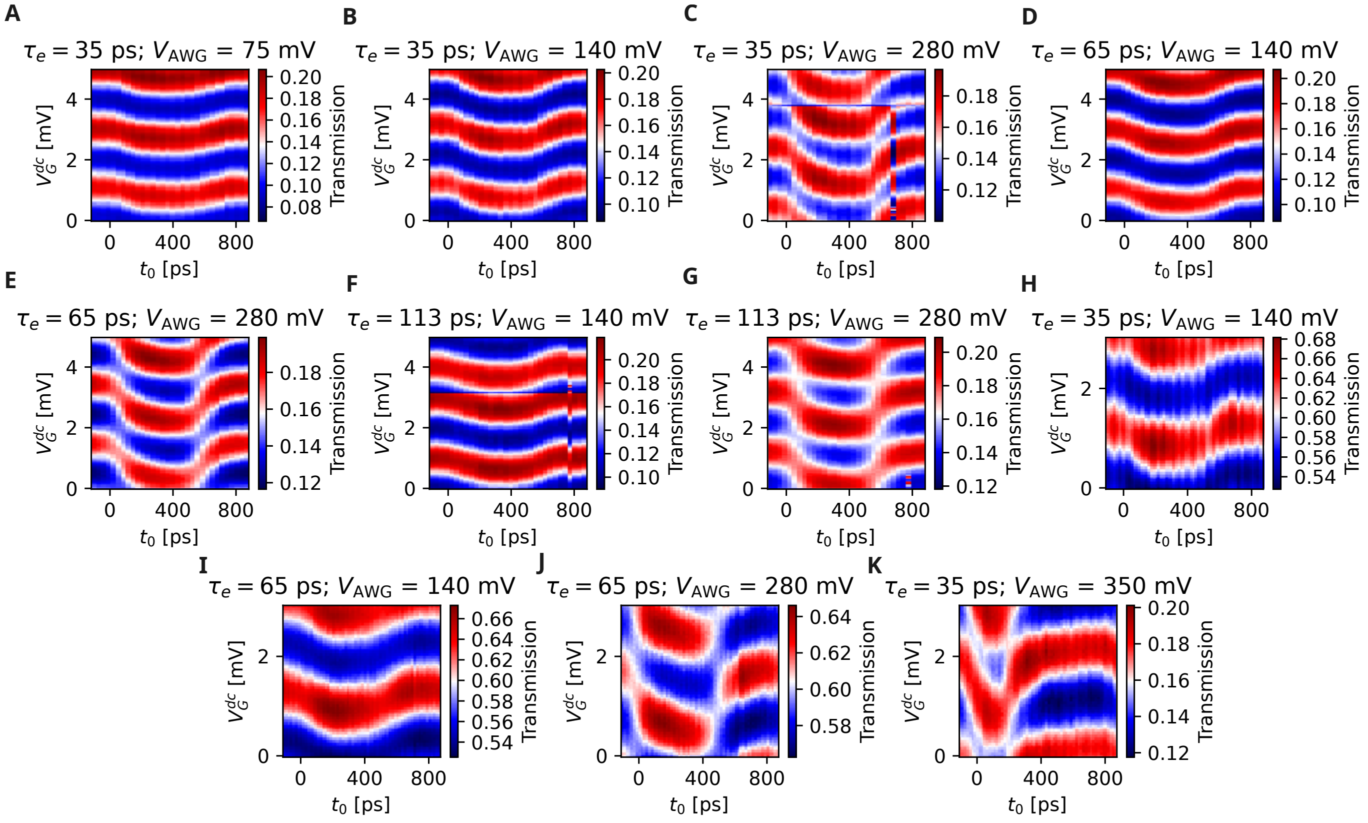

FIG. 7. **Full experimental data set:** (**A**)-(**K**) Time vs gate transmission maps. The pulse widths τ_e and square drive height V_{AWG} are indicated above each map. The data shown in the main text is extracted from maps A, B, C, D, E and G. Maps (**H**)-(**K**) were acquired with a shorter time resolution on t_0. Map (**K**) was obtained by substituting the square drive by a rectangular one of width 250 ps.

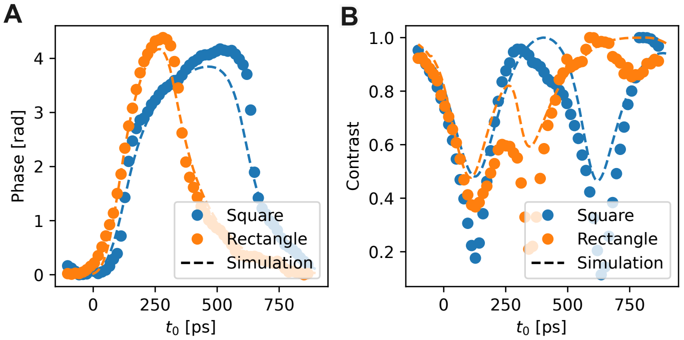

FIG. 8. **Rectangle versus square drive:** (**A**) Phase extracted from maps J (square) and K (rectangle) of figure 7. (**B**) Associated contrast variation. In both graphs, the simulated data is shown in dashed lines.

5. Analysis procedure

In order to extract the phase and amplitude of the interferometric signal, we perform cuts on the two-dimensional maps $T(V_G^{\mathrm{dc}}, t_0)$ (shown in figure 7) at fixed t_0. These cuts show an oscillating signal as a function of V_G^{dc} which is fitted using a cos function of the form $A\cos\left(V_G^{\mathrm{dc}}/V_0 + \vartheta\right) + b$ as shown in figure 10. The fit parameters ϑ and A are then used to plot the main text figures 3G-J. The contrast is then calculated as the ratio $A(t_0)/\max\bigl(A(t_0)\bigr)$.

From these fits we observe that there is no second harmonic contribution to the signal and that a simple sinusoidal oscillation describes our experimental data perfectly, justifying the use of a model where a single round-trip inside the FP cavity is taken into account.

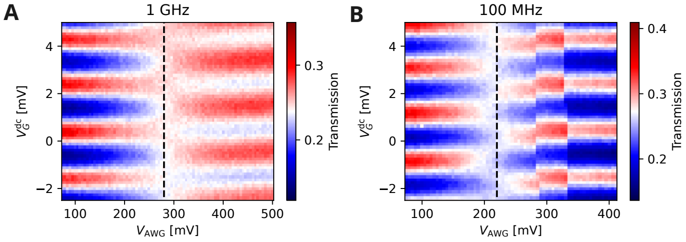

FIG. 9. **Power dependence of the dc transmission:** (**A**) Dependence of the transmission of the FPI as a function of the amplitude of the square drive V_{AWG} and the dc voltage applied on the central gate for a periodic signal at 1 GHz frequency. The vertical dashed line indicates the point where the oscillations disappear at $V_{\mathrm{AWG}} = 280\,\mathrm{mV}$, meaning that the amplitude of the square drive compensates the periodicity of the oscillations along V_G^{dc}. (**B**) Same at 100 MHz. The vertical dashed line indicates the point where the oscillations disappear at $V_{\mathrm{AWG}} = 220\,\mathrm{mV}$.

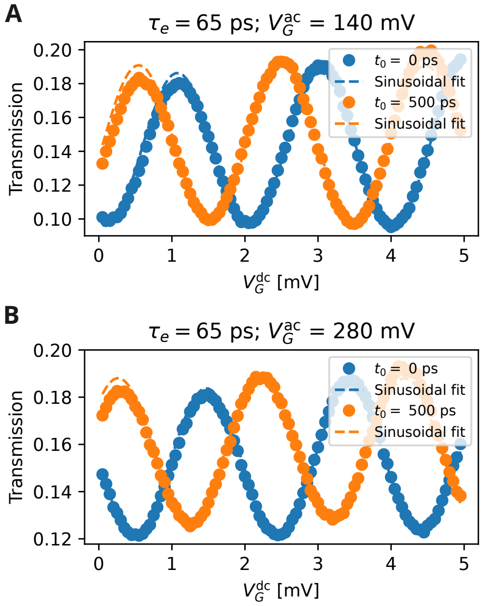

FIG. 10. **Analysis procedure:** (**A**) Dependence of the transmission with V_G^{dc} for $\tau_e = 65\,\mathrm{ps}$ and $V_G^{\mathrm{ac}} = 140\,\mathrm{mV}$ corresponding to data from figure 7D. The two data sets correspond to times $t_0 = 0\,\mathrm{ps}$ (blue) and $t_0 = 500\,\mathrm{ps}$ (orange). They are fitted using a sinusoidal function (dashed lines). (**B**) Same for $\tau_e = 65\,\mathrm{ps}$ and $V_G^{\mathrm{ac}} = 280\,\mathrm{mV}$, corresponding to data from figure 7E.

6. Contrast vs. amplitude of gate oscillations

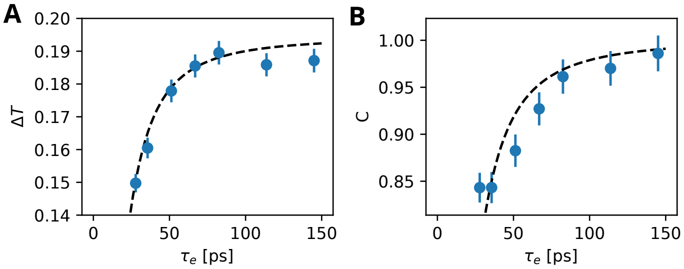

FIG. 11. **Comparison between amplitude and contrast:** (**A**) Extraction of the amplitude of oscillations of $T(V_G^{\mathrm{dc}})$ as a function of the pulse width τ_e. We extract a cavity time $\tau_L = 30\,\mathrm{ps}$. (**B**) Extraction of the contrast of the oscillations (taking into account the varying base line of the data) in V_G^{dc}. The cavity time τ_L =30 ps used for the black dashed line is the same in both figures.

In figures 2B and 2C of the main text, we present the evolution of the contrast as a function of the electronic

temperature and the width of the electronic pulse. We made the choice to plot the contrast instead of the amplitude of the oscillations. However, as we show in figure 11, both quantities lead to the same result when plotted as a function of the pulse width. In figure 11A the blue dots correspond to the amplitude of the oscillations extracted, as explained in appendix E 5. In figure 11B, we present the same data points as in the main text when,instead of the contrast, we plot the amplitude of the oscillations which corresponds to the amplitude divided by the base line offset of the gate dependent oscillations. The contrast is normalized to the value for $\tau_e \to \infty$. In both figures, the dashed line represents the overlap of the wave packets

$$\mathrm{Re}\left[\int \mathrm{d}t \varphi_{\tau_e}(t)\varphi^*_{\tau_e}(t+\tau_L)\right] = \frac{1}{1+(\tau_L/2\tau_e)^2} \tag{E2}$$

from which we can extract the value of τ_L. In figures 11.A and B, the dashed line only differ by a numerical factor in order to rescale this overlap to the quantity that is plotted.

7. Characterization of the QPCs

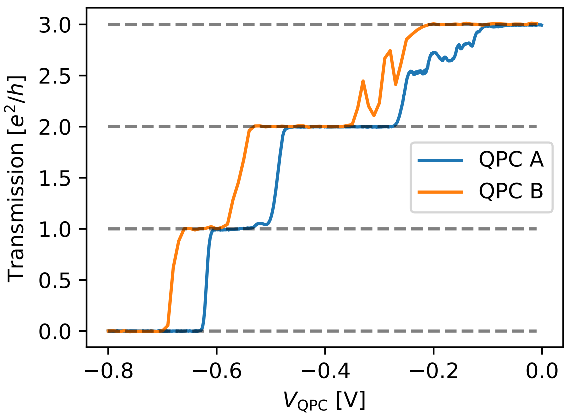

FIG. 12. **Characterization of the QPCs:** Transmission measured through QPC1 and QPC2 as a function of the voltage applied on the QPCs. The dashed line represent the expected position of the quantization plateaus at the filling factor $\nu = 3$.

The data presented in the main text was acquired at the filling factor $\nu = 3$. We characterize the quantization of the Hall plateaus through the transmission as a function of the voltage applied on the QPCs as presented in figure 12. In this figure, we observe three well defined plateaus through both QPCs, quantized at integer values of e^2/h with a maximal conductance of $3e^2/h$, as expected for the filling factor $\nu = 3$.

Appendix F: Numerical modelization

1. Modelization of the square drive

The periodicity of the drive applied to the gate is $2\tau_s = 1\,\mathrm{ns}$. The ideal shape of the drive as sent by the AWG is a perfect square signal, such as the one represented on figure 13 in orange. However, attenuation and capacitances of the cables will deform the signal that is probed at the level of the FPI. We model this signal in our simulation as an exponentially increasing and decreasing wave such as represented in figure 13. This exponential rise is characterized by a rise time τ_r that we take to be equal to 140 ps to better reproduce our experimental data.

2. Continuous evaluation of the parameters

Figure 14 presents the result of the evaluation of the transmission for parameters V_{AWG} and τ_e varying continuously over a larger range than the one shown in the main text. Figures 14.A. and B. respectively show the phase and contrast

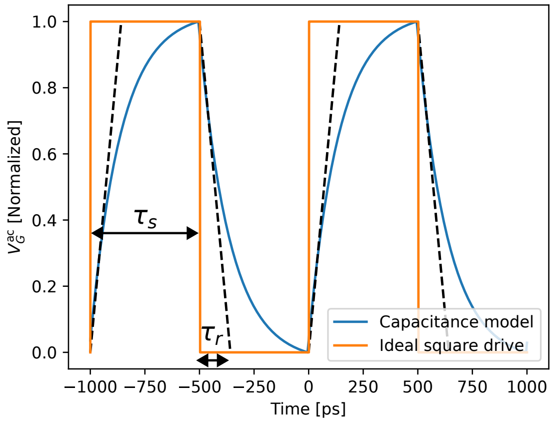

FIG. 13. **Pulse modelization:** An ideal square drive (in orange) can be defined by its periodicity τ_S. Due to various attenuation sources on the rf line used to apply this drive, we expect a deformation of the pulse characterized by a rise time $\tau_r = 140\,\mathrm{ps}$.

for $\tau_e = 35\,\mathrm{ps}$ and amplitudes of the square drive evolving from 28 mV (blue) to 280 mV (red). We can see on panel A that the phase varies linearly with the amplitude of the square drive. Regarding the contrast, the dips around $t_0 = 0$ and $t_0 = 500\,\mathrm{ps}$ become more and more pronounced when the amplitude of the drive increases. As discussed in the main text, the dips of the contrast are related to quantum fluctuations of the phase that increase for increasing amplitude of the square.

Figures 14C and D respectively show the phase and contrast for V_{AWG} =280 mV for continuously varying pulse widths from 20 ps (red) to 130 ps (blue). As we have discussed in the main text, increasing the width of the pulse leads to a reduced phase shift compared to the expected variation for a given amplitude of the square. As seen on panel D, increasing the temporal width of the single electron wavepackets also increases the quantum fluctuations of the phase, leading to more pronounced dips of the contrast at $t_0 \approx 0$ and $t_0 \approx 500\,\mathrm{ps}$.

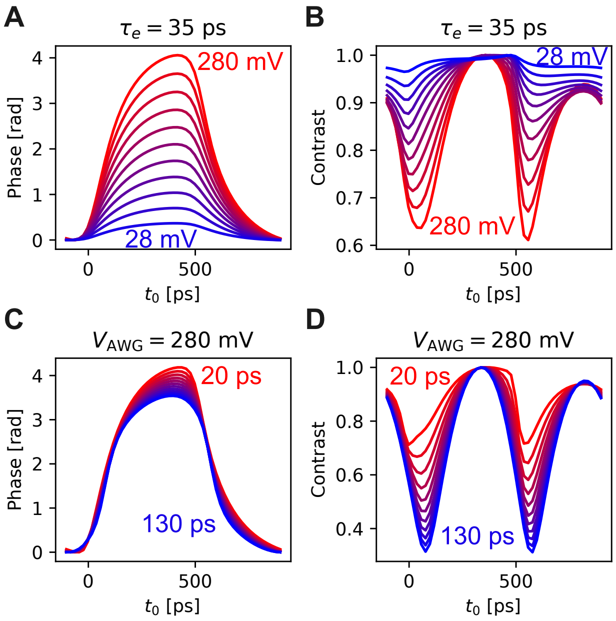

FIG. 14. **Continuous evolution of the parameters:** (**A**) Evolution of the phase calculated for $\tau_e = 35\,\mathrm{ps}$ for voltages V_{AWG} continuously evolving from 28 mV (in blue) to 280 mV (in red). (**B**) Associated contrast. (**C**) Evolution of the phase calculated for $V_{\mathrm{AWG}} = 280\,\mathrm{mV}$ for pulse widths evolving continuously from 20 ps (red) to 130 ps (blue). (**D**) Associated contrast.

Appendix G: Study of the interference pattern in the Fourier space and effects of the Coulomb interaction

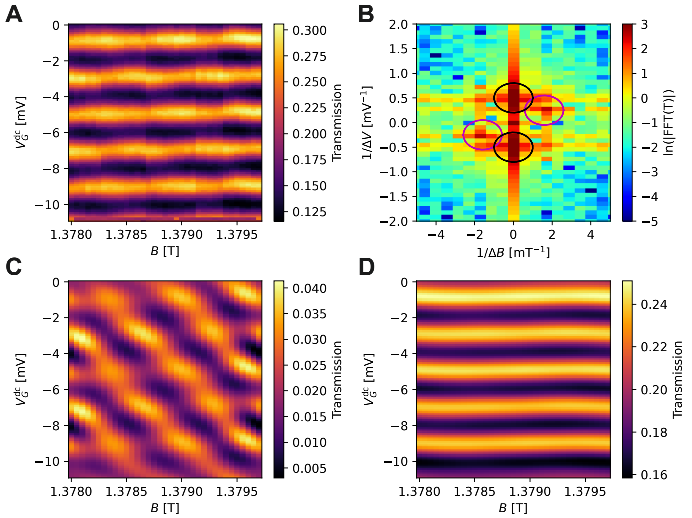

FIG. 15. **Oscillations in Fourier space:** (**A**) Pajama plot of the dc transmission through the Fabry-Perot interferometer as a function of $V_G^{\rm dc}$ and $B(T)$. (**B**) Fourier transform of the signal presented in A. The magenta circle indicates the Aharonov-Bohm contribution to the signal while the black circle indicates that of the Coulomb contribution. (**C**) Filtered Aharonov-Bohm contribution. (**D**) Filtered Coulomb contribution.

Figure 1 of the main text (reproduced here in figure 15.A) shows the oscillating pattern of the transmission of the Fabry-Perot in the $(V_G^{\rm dc}, B)$ space. As discussed in the manuscript, although the B field periodicity matches the expected value for a variation of one flux quantum through the interferometer area, the amplitude of the B oscillations is roughly 5 times smaller than the gate oscillations. As discussed in Ref. [1], this can be explained by interaction effects within the Fabry-Perot cavity. For strong interactions, corresponding to the Coulomb dominated regime, varying the magnetic field leads to a variation of the interferometer area A. This change in A maintains the Aharonov-Bohm phase constant and suppresses completely the variation of T with B when the interfering channel is the outer one [17]. As observed in [3], our device is in an intermediate regime, where Aharonov-Bohm oscillations as a function of B can still be observed yet with a smaller amplitude compared to plunger gate voltage oscillations.

In order to highlight more Coulomb interaction effects and extract the oscillation periodicities more precisely, we perform a two-dimensional Fourier transform of the signal which results in the data shown in figure 15.B. From this procedure, we observe 4 peaks that correspond to two contributions to the interference signal[3]. The first set of peak is the Aharonov-Bohm contribution that has a non-zero contribution in both field and gate voltage and at coordinates $(1.59\,\mathrm{mT}^{-1}, 0.25\,\mathrm{mV}^{-1})$. In voltage and field, these coordinates correspond respectively to periods 0.63 ± 0.22 mT and 4.0 ± 1.4 mV. The second contribution to the signal comes from the bulk-edge coupling by the Coulomb interaction. As a result, it only has a contribution as a function of the gate voltage at coordinates $(0\,\mathrm{mT}^{-1}, 0.5 \pm 0.09\ \mathrm{mV}^{-1})$. This corresponds to a period in terms of gate voltage of 2 ± 0.36 mV. By filtering only these two contributions to the signal we obtain the map shown in figure 15.C. and D. The presence of these two contributions shows that our device sits in the intermediate regime, where both the Aharonov-Bohm and Coulomb dominated periodicities can be observed (with a stronger contribution from the Coulomb dominated regime).

The oscillations as a function of the magnetic field can also be observed in the single-electron regime over a larger field range. We present in figure 16.A a plot of the transmission versus magnetic field (in a field range of of approximately 4 mT) when single electron pulses with a temporal width of $\tau_e = 35$ ps are emitted at the input of the interferometer. By taking the Fourier transform of this trace, we can identify the corresponding Fourier peaks at the same frequency as before $1.58 \pm 0.19\ \mathrm{mT}^{-1}$, that corresponds to a period of 0.63 ± 0.08 mT.

[1] B. I. Halperin, A. Stern, I. Neder, and B. Rosenow, Theory of the fabry-pérot quantum hall interferometer, Physical Review B **83**, 155440 (2011).

[2] H. Souquet-Basiège, B. Roussel, G. Rebora, G. Ménard, I. Safi, G. Fève, and P. Degiovanni, Quantum sensing of time dependent electromagnetic fields with single electron excitations, arXiv:2405.05796 (2024).

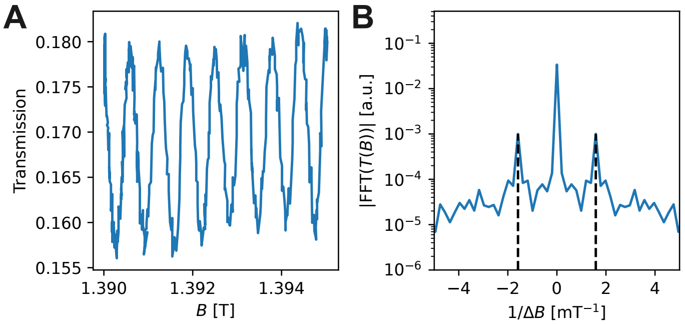

FIG. 16. **Large range field oscillations:** (**A**) Large range oscillations of the transmission of the Fabry-Perot interferometer as a function of the magnetic field. This data was taken in the same configuration as the data in figure 15 but 20 mT higher in field. (**B**) Absolute value of the Fourier transform of A. The vertical dashed lines show the position of the oscillation frequency corresponding to a period $\Delta B = 0.63 \pm 0.08$ mT. The uncertainty corresponds to the resolution of the Fourier space.

[3] I. Sivan, H. Choi, J. Park, A. Rosenblatt, Y. Gefen, D. Mahalu, and V. Umansky, Observation of interaction-induced modulations of a quantum Hall liquid's area, Nat Commun **7**, 12184 (2016).
[4] Note that here and in the rest of this Supplementary, $\varphi_{\tau_e}(t)$ has dimension $[\mathrm{T}]^{-1/2}$.
[5] I. Safi and H. Schultz, *Quantum transport in semiconductor submicron structures* (Kluwer Academic Press, 1995) p. 159.
[6] I. Safi, A dynamic scattering approach for a gated interacting wire, The European Physical Journal B-Condensed Matter and Complex Systems **12**, 451 (1999).
[7] P. Degiovanni, C. Grenier, G. Fève, C. Altimiras, H. Le Sueur, and F. Pierre, Plasmon scattering approach to energy exchange and high-frequency noise in ν= 2 quantum hall edge channels, Physical Review B—Condensed Matter and Materials Physics **81**, 121302 (2010).
[8] P. Degiovanni, C. Grenier, and G. Fève, Decoherence and relaxation of single-electron excitations in quantum hall edge channels, Physical Review B—Condensed Matter and Materials Physics **80**, 241307 (2009).
[9] C. Cabart, B. Roussel, G. Fève, and P. Degiovanni, Taming electronic decoherence in one-dimensional chiral ballistic quantum conductors, Physical Review B **98**, 155302 (2018).
[10] E. Frigerio, G. Rebora, M. Ruelle, H. Souquet-Basiège, Y. Jin, U. Gennser, A. Cavanna, B. Plaçais, E. Baudin, J.-M. Berroir, *et al.*, Gate tunable edge magnetoplasmon resonators, arXiv preprint arXiv:2404.18204 (2024).
[11] A. Delgard, B. Chenaud, U. Gennser, A. Cavanna, D. Mailly, P. Degiovanni, and C. Chaubet, Coulomb interactions and effective quantum inertia of charge carriers in a macroscopic conductor, Physical Review B **104**, L121301 (2021).
[12] Z. Wei, D. Feldman, and B. I. Halperin, Quantum hall interferometry at finite bias with multiple edge channels, arXiv preprint arXiv:2405.05486 (2024).
[13] B. Gaury and X. Waintal, Dynamical control of interference using voltage pulses in the quantum regime, Nature Communications **5**, 3844 (2014).
[14] J.-Y. M. Lee, C. Han, and H.-S. Sim, Fractional mutual statistics on integer quantum hall edges, Physical Review Letters **125**, 196802 (2020).
[15] T. Morel, J.-Y. M. Lee, H.-S. Sim, and C. Mora, Fractionalization and anyonic statistics in the integer quantum hall collider, Physical Review B **105**, 075433 (2022).
[16] Y. M. Blanter and M. Büttiker, Shot noise in mesoscopic conductors, Physics reports **336**, 1 (2000).
[17] N. Ofek, A. Bid, M. Heiblum, A. Stern, V. Umansky, and D. Mahalu, Role of interactions in an electronic Fabry-Pérot interferometer operating in the quantum Hall effect regime, Proceedings of the National Academy of Sciences **107**, 5276 (2010).
